# Starches in Rice: Effects of Rice Variety and Processing/Cooking Methods on Their Glycemic Index

**DOI:** 10.3390/foods14122022

**Published:** 2025-06-07

**Authors:** Muhammad Adil Farooq, Jianmei Yu

**Affiliations:** Department of Family and Consumer Sciences, North Carolina Agricultural and Technical State University, 1601 East Market Street, Greensboro, NC 27411, USA

**Keywords:** rice types, glycemic index, starch composition, starch structure, rice processing, cooking methods, starch–ingredient interaction, gene mutation

## Abstract

Rice is a fundamental food source for more than fifty percent of the world’s population, significantly contributing to human nutrition and food security. Like other cereal grains, rice is rich in starch, although it also contains protein, vitamins, and minerals. Regular consumption of white rice has been reported to be positively associated with the increased risk of type 2 diabetes in rice-consuming countries due to the high glycemic index (GI) of white rice. However, the nutritional value and health effects of rice differ markedly depending on the variety and are influenced by processing methods, cooking styles employed, and the presence of other food components/ingredients. Therefore, this review examines the chemical compositions, starch structures, and glycemic indices of different rice types and the impact of processing techniques and genetic mutation on starch’s structure, amylose content, and GI. The interactions between rice starch and other food components, such as proteins, lipids, dietary fibers, and polyphenols, and their impact on the digestibility and GI of rice starch are also discussed. The purpose of this comprehensive review is to elucidate the strategies that can improve the nutritional advantages of rice and mitigate health issues, such as obesity, diabetes, and inflammation, linked to the long-term consumption of rice.

## 1. Introduction

Rice (*Oryza sativa* L.) is the third most important cereal crops following maize and wheat, and it is also estimated to yield more food energy per hectare than any other cereal crop [1]. Two-thirds of the world’s population uses rice as a staple food. It is reported that world rice production reached 535.8 million metric tons in 2024/2025 as the global population continued to increase [2]. It was estimated that people in Asia obtained 60–70% of their calories from rice and rice-derived products [3]. Rice contains starch as a principal constituent and some minor constituents, such as proteins, lipids, phosphorus, etc. Rice starch is used in various foods and industrial applications as an ingredient, such as baby food, extruded products, soups, dressing, and meat preparation, due to its small granule size and non-allergic characteristic. Despite its nutritional value, rice consumption has been linked to several health issues, particularly in light of rising global rates of diabetes and obesity. The concept of the glycemic index (GI) was introduced to guide diabetics in selecting foods that have low postprandial blood glucose response. Based on the GI, food is often classified as low (less than 55), medium (56–69), and high (70 or more) [4]. On the other hand, other terminology, such as predicted glycemic index (pGI) and estimated glycemic index, is also used to estimate the glycemic effect of foods. The pGI is used to calculate the rate and extent of starch digestion under stimulated gastrointestinal conditions and is commonly derived from the hydrolysis index (HI), while eGI is useful for known GI values by computing the values using empirical or regression-based systems.

The GI of some rice cultivars and an overreliance on refined white rice have prompted concerns about its role in metabolic diseases. Rice is often classified as a high- GI food due to its high starch content, which makes it unsuitable for those suffering from diabetes or other metabolic disorders [5,6]. However, the GI values of rice are quite diverse and can vary widely [7,8,9]. Rice’s GI value is impacted by a wide range of variables, such as genetic, environmental, and processing-related factors [8,10,11,12]. The International Rice Research Institute (IRRI) and the Commonwealth Scientific and Industrial Research Organization (CSIRO) Food Futures Flagship conducted a comprehensive study on 235 rice varieties worldwide. The results showed that the GI varied greatly, ranging from 48 to 92 (average value 64), in contrast to glucose (100) [13].

**Figure 1:**
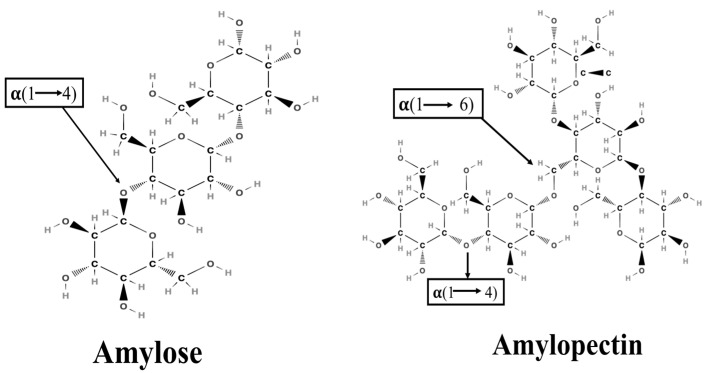
Molecular structure of amylose and amylopectin.

Starch is a white crystalline powder. It comprises two polysaccharides: amylose and amylopectin. Amylose is a linear chain of glucose molecules joined by α-1,4 glycosidic bonds, while amylopectin is a highly branched polymer composed of glucose monomers joined by α-1,4 and α-1,6 glycosidic bonds (Figure 1) [14]. Amylose, characterized as a linear starch molecule, exhibits a denser and more organized structure than amylopectin, a branched starch molecule. The compact structure of amylose reduces its accessibility to digestive enzymes, decelerating starch digestion [15]. Amylose can recrystallize into resistant starch, which is resistant to enzymatic breakdown in the gastrointestinal track [16]. Rice varieties containing a higher amount of amylose content are less vulnerable to enzymes and show lower digestibility than amylopectin-rich varieties [17]. Different factors, such as the amylose-to-amylopectin ratio, types of rice, processing methods, and non-starch constituents (lipids, proteins, dietary fibers, and polyphenols), significantly affect the rate of hydrolysis/digestion [18]. According to the rate of digestion, starch is classified as rapidly digestible starch (RDS), slowly digestible starch (SDS), and resistant starch (RS). RDS is rapidly digested in the GI track and absorbed in the small intestine, thus resulting in a quick increase in blood glucose levels, while SDS is digested more slowly and promotes better glycemic control. RS is resistant to digestion and can be fermented in the colon to form short chain fatty acids, providing advantages for gut health. On average, RDS, SDS, and RS account for 70–80%, 5–10%, and 1–10%, respectively, of cooked starchy foods [19,20].

Rice processing and cooking methods and the interaction between starch and other food components significantly affect the digestibility of starch, thus affecting the GI value and nutritional properties of rice. Understanding these interactions is critical for creating rice-based products that address specific dietary needs and health goals. This review article seeks to provide a comprehensive understanding of factors, such as rice variety and cooking methods or processing techniques, including milling, extrusion, steaming, boiling, and baking, that impact the chemical makeup, starch granule structure, and digestibility of rice starch, thus affecting the GI of rice. By summarizing current research, this review aims to provide insights into enhancing rice-based diets to address global health challenges associated with rice consumption.

## 2. Starch Composition and Granule Structure of Rice

Cereal starch consists of amylose and amylopectin, which vary in branching structure and molecular weight. The chemical structure of starch is complex and arranged into six hierarchical levels, encompassing individual glucose chains and their interactions with other grain constituents, such as proteins and lipids. This hierarchical structure affects starch’s physical and functional characteristics, including digestibility and texture.

**Figure 2:**
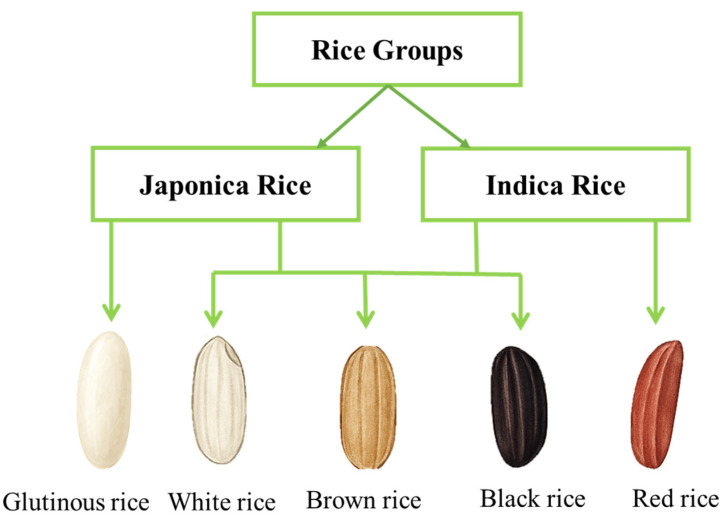
Representative types of rice. White, brown, and black rice fall into the groups Japonica and Indica. The red rice belongs to Indica due to its non-sticky nature. The glutinous rice was placed into the Japonica group due to its low amylose content and sticky nature.

Rice possesses various functional characteristics due to different amylose and amylopectin ratios. When rice is cooked, amylose, which typically accounts for 15–30% of the starch, produces a firmer and less sticky texture. In contrast, amylopectin, which makes up 70–99% of rice starch, has a highly branched structure that gives cooked rice a cohesive and sticky texture [21,22]. The amylose and amylopectin content of rice varies with rice varieties, which are generally classified into two subspecies: Indica and Japonica (Figure 2). Indica varieties tend to have higher amylose content (20–30%), resulting in a stiffer and less sticky texture of cooked rice. Conversely, Japonica cultivars have a lower amylose concentration (10–20%), which leads to a softer and stickier texture [23]. Moreover, the digestibility of rice is also influenced by its amylose content. According to Boers et al., starches with a higher amylose level are more difficult to digest because amylose forms tighter, more compact structures that are challenging for digestive enzymes to break down [24]. As a result, rice varieties with higher amylose content, such as Basmati, generally have a lower GI, which can benefit blood sugar regulation.

Digestibility is significantly influenced by the physical structure of rice starch granules and their chemical composition [25]. Granules of rice starch are usually polyhedral and tiny (2–8 μm), exhibiting a characteristic A-type crystalline structure. Rice starch is more prone to enzymatic degradation than B-type starch found in tubers. The surface characteristics of the granules, such as their pores and channels, affect the ease with which the starch molecules are accessed by digesting enzymes, such as amylase [26]. Certain rice types are slowly digested and reduce the glycemic effect due to their more compact granule shapes with fewer surface imperfections. Furthermore, granule integrity can be changed by processing techniques like grinding and parboiling, which can either increase enzyme accessibility (raising the GI) or cause structural alterations that improve resistance to digestion (reducing the GI) [27].

## 3. Rice Types and Glycemic Index

**Figure 3:**
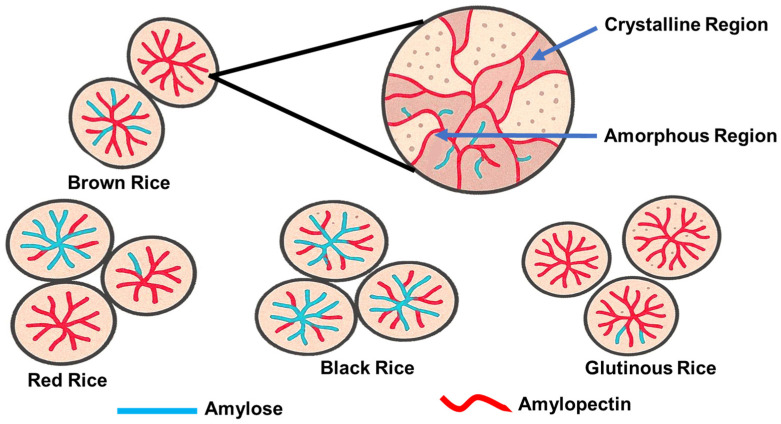
Granular structures of native rice starches from different rice varieties or cultivars to show the amylose/amylopectin ratios.

More than 40,000 varieties of rice has been reported, and over 90,000 samples of cultivated rice and wild species are stored at the International Rice Gene Bank [28]. However, only a few varieties are cultivated extensively for human consumption. White rice is the most consumed worldwide; some specialty rice cultivars, such as colored and aromatic rice, are also grown and marketed [29], but in much smaller quantities. The contents and composition of starch in rice vary significantly across different rice types and degrees of milling. Table 1 and Figure 3 shows that white rice typically contains more starch than naturally colored rice, and refined rice has higher starch content than less processed rice, such as brown rice, due to the removal of the bran and germ. Among all types of rice, glutinous rice has the highest starch content, and the starch in glutinous rice is mainly amylopectin [23,30], while the starch in other types of rice contains 20–25% amylose and 70–80% amylopectin.

Based on amylose content, rice is classified as waxy (0–2% amylose), very low amylose content (2–12%), low amylose content (12–20%), intermediate amylose content (20–25%), and medium to high amylose content (20–33%) [21]. Rice cultivars with more amylose content have a lower GI due to longer starch digestion, but those with higher amylopectin content have a higher GI due to faster glucose release. A study examined 25 genotypes of Japonica rice with 5–25% amylose concentration and found that their glycemic index (GI) varied significantly [31]. Ten of these genotypes had a medium GI, ten had a high GI, and five had a low GI. Additionally, the study found a negative correlation (r = −0.528) between medium GI and amylose content, suggesting that lower GI values are associated with higher amylose levels. Gunaratne et al. [32] evaluated and compared the digestibility rates, estimated glycemic index (eGI), gelatinization properties, and pasting properties of six popular high-amylose rice varieties cultivated in Sri Lanka, and the results showed that all freshly cooked rice samples had a narrow range of starch digestibility and eGI, ranging from 88.2 to 92.4. Furthermore, the amylopectin chain length also affects the GI of carbohydrate foods. The researcher found a lower GI in the starches containing shorter amylopectin chains, while those with higher GI values were linked to longer chains [33].

**Table 1:** Starch contents, compositions, and glycemic index of different types of rice.

Rice Type	Starch Content (%)	Amylose (%)	Amylopectin (%)	Glycemic Index	Reference
White Rice	70–80	20–25	75–80	64–93	[34]
Brown Rice	65–75	20–25	75–80	50–55	[35]
Red Rice	65–75	20–30	70–80	55–65	[36,37]
Black Rice	60–70	15–20	80–85	42–50	[38,39]
Glutinous Rice	75–85	0–5	95–100	75–98	[23,40]

### 3.1. White Rice

White rice is obtained by polishing hulled rice. Polishing alters rice grains’ nutritional composition by removing the grain’s outer layers, including the pericarp, seed coat, testa, aleurone layer, and embryo [41]. White rice is the most commonly consumed among the different rice varieties due to its palatability. It is easy to cook and has a better shelf life than its counterparts. However, the polishing process significantly reduces the nutritional value of rice because the components removed by polishing are the main source of dietary fiber, lipid, protein, B vitamins, minerals, and other antioxidants [42]. White rice possesses a high GI, which is associated with worsening glucose intolerance in countries where white rice is used as a primary staple food. More importantly, a fast increase in diabetes incidence has been reported in these areas [43].

Further research has revealed geographical variances in rice’s GI. A study examined the GI of twenty rice varieties grown in India and found that amylose content ranges between 21.7% and 24.73%, and their GI ranges between 56 and 69 [44]. Another study conducted in Malaysia reported a high GI value in fragrant white rice (FWR) compared to white rice (WR), which was 5% broken grains [45].

### 3.2. Glutinous Rice

Waxy rice, also known as sticky rice or glutinous rice (*Oryza sativa* var. *glutinosa*), is a unique type of rice characterized by its high concentration of amylopectin. According to Setyaningsih et al. [46], glutinous rice contains 98–100% of amylopectin by dry weight, which sets it apart from other rice varieties and gives it a distinctly sticky and chewy texture after cooking [47]. Most glutinous rice is cultivated in Southeast, East, and certain regions of Asia, with countries like Thailand, Laos, Vietnam, and China accounting for a significant portion of global production. Despite its widespread consumption and cultural importance, glutinous rice has a high GI, typically ranging from 77 to 99 [48]. This high GI is attributed to its near-exclusive amylopectin content, which is quickly converted to glucose during digestion, leading to a surge in blood sugar levels [49]. As a result, regular consumption of glutinous rice has been linked to an increased risk of insulin resistance and type 2 diabetes [50]. This poses particular health concerns in regions where glutinous rice is a staple food and consumed in large quantities. Sheng et al. [51] reported low amylose content (1.93%) alongside higher amylopectin content (98.57%) and pGI values (85.44%) for glutinous rice starch when compared to Indica rice starch (IRS), which has higher amylose content (24.11%) and lower amylopectin content (76.88%) and pGI values (81.16%). Similarly, another study chose rice varieties based on their apparent amylose (AA) content and resulted in lower levels of RS at 0.15% and an eGI of 95.8% in white cooked waxy rice, while cooked brown waxy rice had 0.24% RS and 76.4% eGI compared to cooked non-waxy rice starches [52]. This reveals that rice’s amylose content is crucial in determining its GI, with more significant amounts resulting in slower digestion and a lower GI.

The GI of glutinous rice is also affected by multiple factors, such as protein, lipids, the digestion rate, and processing conditions. For example, parboiling or partially boiling the rice in its husk is one way to lower the GI of sticky rice. Parboiling changes the starch structure, resulting in the development of amylose–lipid complexes. These complexes digest more slowly in the small intestine, lowering the rice’s glycemic index [53]. In addition to its carbohydrate content, sticky rice bran is a rich bioactive chemical source with numerous health benefits. Antioxidants found in bran include γ-oryzanol, γ-tocotrienols, ferulic acid, phytosterols, tocols, triterpenic alcohol, fibers, and unsaturated fatty acids. These substances have been demonstrated to exhibit hypolipidemic (lipid-lowering) and hypocholesterolemic (cholesterol-lowering) actions in humans [54]. The γ-oryzanol and phytosterols, for example, can limit cholesterol absorption in the gut, whereas unsaturated fatty acids, such as oleic and linoleic acid, promote heart health. Consuming glutinous rice bran has been demonstrated in studies to have cardio-metabolic benefits for obese, diabetic, hypercholesterolemic, and even healthy people [55].

### 3.3. Brown Rice

Brown rice is dulled regular rice with bran remaining. Brown rice has a unique starch structure that remains in the bran, reducing water diffusion and making it difficult for amylase to effectively break down the starch [56,57]. Rice bran is composed of dietary fiber (20.5–33.3%), starch (16.1–26.7%), ash (9.2–13.9%), protein (13.2–18.6%), and lipid (9.5–22.9%) [58]. By interacting with starch or creating protective barriers, proteins in brown rice further slow starch digestion, and lipids and polyphenols reduce starch swelling by building complexes with amylose [59]. Heat treatments like hot air and steam cooking encourage the production of V-type amylose–lipid complexes, even though high pressure and superheated steam can boost starch digestibility by breaking down its structure [60,61,62]. After cooking, cooling improves amylose chain interactions, further slowing digestion. Because of these mechanisms, brown rice has a reduced GI, which is advantageous for controlling blood sugar levels and maintaining metabolic health.

In vivo studies reported that brown rice has a lower GI and better health results than refined white rice because it digests more slowly [63,64,65]. Several factors contribute to this delayed digestion: (1) increased dietary fiber, protein, and amylose content in brown rice [66,67], (2) the presence of bran, which restricts water absorption and keeps starch granules from swelling, reducing their accessibility to digestive enzymes [68], and (3) increased phytic acid, polyphenol, and oil content, which further decreases starch digestibility. Phytic acid has been demonstrated to reduce the GI [69], and polyphenols block the enzyme α-amylase, which breaks down starch [70]. With its lowest GI, brown rice is especially advantageous for people trying to regulate their blood sugar levels compared to polished white, red, and black rice [64]. According to an in vitro study, white rice digests more readily than brown rice, as shown by the fact that it becomes less hard after digestion [71]. Similar to this, white rice flour releases more maltose and digests more quickly than black or brown rice flours, which are slower to digest because of their higher protein and polyphenol content [72,73]. According to Farooq et al. [74], brown rice flour has a lower pGI because of its larger starch granules, which also lessen swelling and hydrolysis. According to these results, rice’s structure and bran content affect its GI and digestibility.

### 3.4. Black Rice

Black rice is abundant in vitamins, minerals, fiber, and plant-derived proteins, with phenolic compounds, such as anthocyanins, tannins, phenolic acids, and flavonoids, enhancing its health advantages [75,76]. It is devoid of cholesterol, low in sugar and fat, and rich in phenolic compounds, dietary fiber, and antioxidants, rendering it a better substitute for white rice [77,78]. The reduced GI results from the development of amylose–lipid complexes during gelatinization, which enhances resistant starch (RS) and slowly digested starch (SDS) while reducing rapidly digestible starch (RDS) [79]. An et al. [80] found that replacing 10–40% of wheat flour with black rice flour decreased glucose release during digestion. Black rice flour is a promising food ingredient for controlling blood sugar, especially for people managing or at risk for diabetes, because of these qualities. In addition to being high in macronutrients, black rice flour is also a great source of phytochemicals, particularly anthocyanins, which are the natural pigments that give it its vibrant color, and other polyphenols [81,82,83,84,85]. The bran has the highest concentration of these substances, followed by the whole grain, endosperm, and husk [86], and they are known for their antioxidant qualities and aid the body’s fight against free radicals [83,87]. These anthocyanins slow down starch digestion by inhibiting the activity of amylase and α-amyloglucosidase [88,89]. These characteristics, as mentioned earlier, render black rice bran an exceptionally potent component for augmenting the nutritional and health advantages of food items, specifically for regulating glycemic reactions and offering antioxidant reinforcement.

### 3.5. Red Rice

Red rice is a type of rice that has not been polished and has kept its red bran layer. It is abundant in anthocyanins, minerals, and vitamins [90]. The presence of beneficial phytochemicals, such as tocopherols, tocotrienols, γ-oryzanol, and phenolic components, in this substance renders it exceptionally nutritive [91]. The pericarp of pigmented rice, which includes both light and dark kinds, contains anthocyanins that give it a characteristic dark crimson or red hue [92]. The nutrient density, beneficial qualities, and resistance to milling make them valuable. There are more than 300–400 types of red rice [93], each with unique culinary and nutritional characteristics [94]. Due to their medicinal properties, which include the ability to treat cancer and diabetes, many of these cultivars are utilized in Ayurvedic medicine [95]. Antioxidants and dietary fiber, especially in red rice, can lower the glycemic index (GI) and minimize the risk of type 2 diabetes [38]. A recent study examined 12 types of rice based on their colors collected from different areas of China [96]. The results showed that Ningbo’s glutinous white rice had the highest GI, while colored rice, such as black and red rice, mainly from Suihua, had the lowest. Crystallinity, composition, and starch concentration were also important factors influencing GI. Another study examined the impact of purple red rice bran anthocyanin extracts (PRRBAE) on the physicochemical properties and digestibility of rice starch and found the formation of V-type complexes with increased antioxidant activity, elevated RS concentration, and inhibited enzyme activity [97]. These effects demonstrate PRRBAE’s potential for producing high-nutritional-value and low-GI food products.

## 4. Effects of Food Ingredient Interaction on Rice Starch

The interaction of food components with rice starch determines their functional and nutritional qualities. Starch–protein interactions frequently result in the formation of complexes, which can improve gel flexibility and texture while potentially lowering starch digestibility [98]. Starch amylose–lipid interactions produce complexes that limit gelatinization and retrogradation, extending shelf life and increasing texture in rice-based baked goods [99]. Starch–dietary fiber interactions can improve water retention and gel stiffness while decreasing starch digestion, resulting in a lower GI [100]. Finally, starch–polyphenol interactions, generally through non-covalent bonding, can change starch structure and digestibility, reducing enzymatic hydrolysis and influencing the color and aroma of the finished product [101]. These interactions affect rice-starch-based meals’ physicochemical qualities, mouthfeel, and nutritional value. Table 2 presents the interactions between rice starch and proteins, lipids, dietary fibers, and dietary polyphenols of different origins, as well as the mechanisms and outcomes.

### 4.1. Starch–Protein Interaction

Black, brown, and red rice varieties have more protein in their bran; therefore, they have a higher protein/starch ratio than white rice. Endosperm and bran of rice contain protein [101]. Despite having a low protein content, the protein found in starchy crops like rice interacts with starch and creates complex systems, including composites, gels, and mixtures [107,121]. These interactions may also affect the rate at which starch is broken down and absorbed, which may influence the crop’s overall glycemic and insulin-emic response. A study reported that protein hydrolysis and denaturation could restrict starch hydration and enzymatic cleavage, which was evidenced by differential scanning calorimetry and confocal microscopy [122]. Moreover, protein can also modulate digestion by interfering with metabolic enzymes responsible for starch hydrolysis. It has been found that endogenous lipids and proteins reduce the GI in Indica rice varieties [105]. Additionally, they restrict the swelling of starch granules, most likely by encasing them in a protective layer that prevents the carbohydrase from accessing glycosidic bonds.

Protein-rich pulses can impede starch digestion by forming physical and chemical barriers (steric hindrance and disulfide linkages). Ai et al. [123] discovered that adding protein-rich pulses to a starch-based system (e.g., rice or wheat) disrupted the natural interaction of starch molecules with the proteins already present in the food matrix. Kumar et al. [108] investigated the effect of combining rice with various pulses. They discovered that pigeon pea was more successful in decreasing the GI than other pulses.

Starch and protein interaction greatly influence the gelatinization and retrogradation properties of the rice and rice-based products [124,125]. This interaction can change rice’s behavior during cooking, storage, and consumption by changing its texture, stability, and quality. Rice proteins and the free amino acids released during digestion have been shown in earlier studies to substantially impact the digestibility of rice starch. For example, research has demonstrated a negative relationship between in vitro starch digestibility and the amount of residual protein in rice; slower starch digestion is linked to higher residual protein levels [103]. Another study discovered that hydrolyzed pea proteins and rice protein hydrolysates significantly lowered wheat and rice starch’s digestibility [107,126]. Chi et al. [107] provide more in-depth explanations of the mechanics underlying this phenomenon.

Lu et al. [104] found that rice proteins’ free amino acids inhibit α-amylase, and, when mixed with starch through moisture-heating treatment, the slowly digested starch (SDS) increased from 29.6 to 41.3 g/100 g. This enhances the starch structure and reduces digestion, benefiting low-GI food formulation. The viscosity and enthalpy of gelatinization in rice starch gradually decreased as the amount of rice protein increased, according to research by Wu et al. [127] indicating a slower gelatinization process; the gelatinization temperature rose concurrently. In a comparable investigation, Baxter et al. [128] found that adding 1–10% rice glutenin to rice starch reduced the peak and final viscosity. Khatun et al. [103] found that residual protein levels negatively correlated with digestibility, while adding specific proteins (e.g., 2.0% albumin, 5.0% glutelin) reduced it by 6.3–10.7%. Acetic-acid-extracted proteins also decreased starch digestibility dose dependently. Adjusting protein content or composition through breeding or pre-cooking additions can manipulate starch digestibility and its predicted glycemic index.

### 4.2. Starch–Lipid Interaction

Rice is a high-starch, low-lipid crop. About 0.3–4% lipid is present in white rice flour. Lipids are present in the form of lysophospho lipids, monoacyl lipids, and free fatty acids (FFAs inside of the starch granule) and as phospholipids, glycolipids, triglycerides, and glycolipids (on the granule’s surface) [129]. The addition of lipids (oil or fat) is common during rice cooking/preparation. Starch and added lipids can interact with each other through non-covalent interactions, such as hydrophobic interaction, van de Waals forces, or hydrogen bonding, to form a complex with a more compact V-type structure that is resistant to enzymatic digestion, thus reducing the GI of starchy food [130]. For example, fats, such as soybean oil or ghee (clarified butter), are frequently included with rice in traditional cooking methods, which can change the rate at which the starch breaks down [131]. In laboratory testing, it has been demonstrated that adding these oils to brown or milled rice, whether before, during, or after cooking, slows down the pace at which starch is digested. In particular, when ghee was added before or during brown rice’s boiling, the least amount of glucose was released after digestion [131]. The addition of palm oil to brown, black, milled, and waxy rice increased the quantity of RS while decreasing the levels of SDS and RDS [131,132]. However, the palm oil’s addition did not affect the digestibility of waxy rice. In a study by Chen et al., rice flour and starch were cooked at 20%, 30%, and 40% moisture content after mixing with maize oil [99]. Results showed that the oil increased the amounts of SDS and RS and decreased RDS in both flour and cooked rice starch, and the highest amounts of SDS and RS were found at a moisture content of 20%.

### 4.3. Starch–Dietary Fiber Interaction

Dietary fibers (DFs) are non-digestible carbohydrates comprising various substances with different molecular sizes, chemical compositions, and physical and chemical characteristics. They are classified based on their water solubility. The soluble DF includes pectin, beta-glucan, and gums, while insoluble DFs consist of cellulose, hemicellulose, and lignin. The starch–fiber interaction is vital, as it significantly reduces the GI by increasing RS content and restricting enzyme hydrolysis [133,134]. For example, the enrichment of rice grain with soybean dietary fiber and protein significantly increased the soluble dietary fiber, ranging from 0.0021 g g^−1^–0.216 g g^−1^, and decreased rice digestibility and GI value in extruded rice with 6–15% fiber content [135]. Similarly, a study reported an increase in the RS content after adding FiberCreme (an increase in total fat content and a decrease in the total carbohydrates), leading to decreased GI in human trials [112].

Rice polishing eliminates the grain’s DF-rich outer layers, leaving only the endosperm high in starch. White rice has only 0.5–2% DF, but unpolished rice has about 6%. Research has continuously demonstrated a clear correlation between frequent white rice consumption and an increased risk of type 2 diabetes [39]. It has been shown that increasing the fiber content of white rice lowers its GI, which may reduce the risk of type 2 diabetes [115]. One noteworthy invention found that high-fiber white rice (HFWR) has a medium GI (61.3 vs. 79.2) because it has five times as much fiber (8.0 vs. 1.58%), 6.5 times as much RS (3.9 vs. 0.6%), and more amylose (32.8 vs. 26.0%) [115]. HFWR is a healthier option than regular white rice because of its 23% lower GI. In the presence of sufficient amounts of DF, the DF acts as a physical barrier that reduces the accessibility of starch molecules to digestive enzymes due to its high viscosity, physical embedding, and cell wall integrity [136]. DF can restrict heat transfer, water ingress, and starch granule swelling, potentially inhibiting gelatinization and promoting starch retrogradation [137]. All of these will slow down the glucose release in the gastrointestinal tract, thus lowering the GI of starch. Therefore, preparing or consuming rice with a sufficient quantity of DF can prevent the surge of glucose levels in the blood, which is extremely important for patients with diabetes.

### 4.4. Starch–Polyphenol Interaction

Polyphenols are plant secondary metabolites with one or more hydroxyl groups. They are divided into four classes according to their structure: lignans, stilbenes, flavonoids, and phenolic acids [138]. In recent years, more attention has been paid to how various polyphenols impact the digestibility and GI of rice flour, rice starch, and rice-based products [100,139]. They slow starch digestion by first blocking enzymes like amylase, which convert starch into simple sugars. Secondly, they interact with starch molecules to create complexes with V-type crystalline structures, which could become more resistant to enzymatic degradation due to these compounds [100]. Consequently, they raise the amounts of RS and SDS, which are both positive for gut health and blood sugar regulation. Hydrogen bonds and van der Waals forces are the driving mechanisms for the interaction between starch and polyphenols.

According to a study by Meera et al. [64], rice with more phenolic compounds also tends to have a lower GI. According to Aalim et al. [140], phenolic compounds found naturally in black rice worked better than dietary fiber at delaying starch digestion. This demonstrates the significance of phenolic substances in creating starch-based healthy diets that, by controlling blood sugar levels, can aid in managing metabolic diseases like diabetes. Cooked rice containing polyphenols derived from butterfly pea flowers slowed down starch digestion in in vitro studies without affecting customer acceptance [141]. Recent research by El Oirdi [142] demonstrates that polyphenols can reduce the GI of foods both in vitro and in vivo, underscoring their potential for blood sugar regulation. Consequently, purple rice (*Oryza sativa L. indica*) and other colored or pigmented rice types are becoming increasingly popular because of their low GI values compared to white rice and their potent antioxidant and antidiabetic activities [143,144]. Polyphenol-rich rice-based foods provide consumers with a great gluten-free and health-conscious choice. Consuming colored rice, well-known for its antioxidant potential, may also lower cancer risk [100,144].

While most studies found that starch–polyphenol interactions significantly reduce the starch hydrolysis rate, few studies reported no effect on the hydrolysis rate of gelatinized starch after interacting with polyphenols [145,146], because adding higher concentrations of polyphenols makes starch’s structure loose and more porous, allowing the enzymes to easily penetrate the starch [146]. However, the polyphenols used in these studies were ferulic acid and quercetin, whereas anthocyanins and proanthocyanidins are the major polyphenols in colored rice [144]. Thus, comparative research under standardized conditions (e.g., comparing different polyphenol types, concentrations, and rice varieties) is needed to better understand potential inconsistencies. Whether extrinsic (added during processing or cooking) or intrinsic (found naturally in rice), these polyphenols can bind with starch molecules and inhibit the activity of digestive enzymes, thus slowing down the conversion of starch to glucose. In addition, the polyphenols also interact with proteins and lipids through covalent and non-covalent binding, which reduces the digestibility of these nutrients [147]. Therefore, when applying starch–polyphenol interaction strategy to reduce the GI of rice, it is important to select a type of polyphenol with minimal impacts on the digestion and absorption of other essential nutrients in the gastrointestinal tract.

## 5. Rice Genotypes

**Figure 4:**
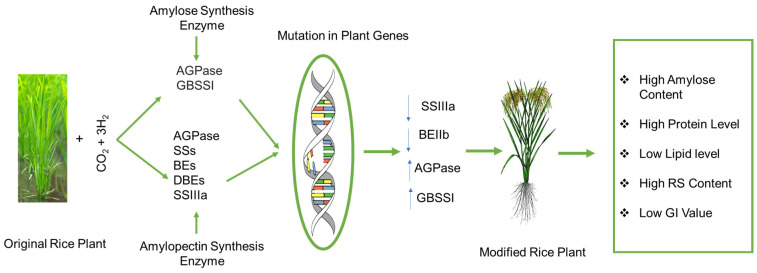
Proposed mutation in rice plant genes by reducing SIIIa and BEIIb levels and forming high RS rice genotypes with low GI. AGPase (ADP-glucose pyrophosphorylase) and GBSSI (granule-bound starch synthase I) play important roles in the formation of amylose and amylopectin synthesis enzymes, including AGPase (ADP-glucose pyrophosphorylase), (SSs) starch synthases, (BEs) branching enzymes, (DBEs), (SSIIIa) debranching enzymes, and starch synthase IIIa (a major isoform of SSIII). Through mutation in plant genes, amylose content increases with increasing AGPase and GBSSI enzymes while decreasing the large branching of amylopectin by reducing the starch synthase IIIa and branching enzymes (DBEs).

Rice’s starch synthesis is a complex process requiring the coordinated actions of several regulatory factors and enzymes (Figure 4). These enzymes often work as multi-enzyme complexes, which means they communicate and work together to produce starch effectively [148]. Starch biosynthesis may also be influenced by non-enzyme factors, such as starch-binding domain-containing protein 1 (SBDCP1), which interacts with starch synthase [149].

Numerous genes are essential modulators of RS and SDS content in rice. For example, the high RS concentration in the Chinese rice variety “Jiangtangdao1” was discovered to be caused by a mutation in the BEIIb gene [150]. RS and RDS levels have been associated with the Wx gene, which codes for granule-bound starch synthase, a potential gene affecting RS content in a genome-wide association study that included 106 different types of rice [151,152]. Additional genes linked to controlling RS content include SSIIa, ISA1 (isoamylase I), and AGPS1 (ADP-glucose pyrophosphorylase small subunit 1 [152].

Moreover, SSIIIa (starch synthase IIIa) is recognized as a key gene that causes RS production in rice. However, its impact on RS was reported to be contingent on the existence of the Wx gene. In particular, the elevated RS level was caused by a faulty SSIIIa gene in conjunction with excessive Wxa expression [153]. Furthermore, it has been demonstrated that mutations in BEIIb and its double-mutant lines, especially those with decreased SSI activity, dramatically raised the amount of RS [154]. CRISPR-Cas9 is a powerful gene editing technology also used to introduce specific mutations in the SBEIIb gene (starch branching enzyme IIb) inside of an elite low-glutelin japonica rice cultivar, resulting in high-resistant starch (RS) and low-glutelin sbeIIb/Lgc1 lines. The protein and starch composition of the mutant lines changed significantly; the RS increased by 6%, and the amylose content doubled, while the glutelin levels remained low. Recently, Badoni et al. [155] created recombinant inbred lines from Samba Mahsuri and the IR36 amylose extender (IR36ae) to produce low-GI rice types with protein content greater than 14%.

## 6. Processing Effects on the Glycemic Index of Rice

**Figure 5:**
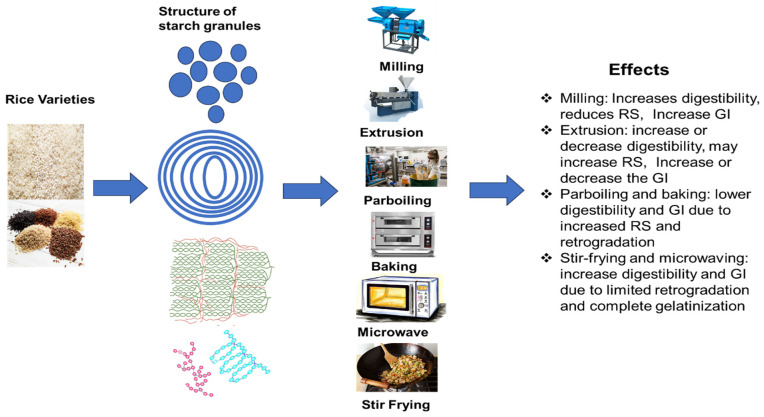
Schematic diagram of the impact of different processing methods on the starch properties and glycemic index of rice starches. Blue color revealed starch granule and layer structure. The green color indicated starch crystal structure, while amylose appeared in a sea green color and amylopectin was represented in a dark pink color.

Processing methods also influence the digestibility and glycemic index of rice. The processing of rice profoundly influences its starch composition, structure, and digestibility, as shown in Figure 5 and Table 3. Milling, extrusion, and heating often elevate the GI. In contrast, techniques, such as partial milling, retrogradation, and incorporating additional ingredients, can augment RS and reduce the GI, enhancing the nutritional quality of rice-based products. For example, Kongkachuichai et al. [156] demonstrated that paddy processing techniques significantly affected rice’s GI value and bioactive components, especially in Thai landrace varieties. The study found that polishing significantly lowered the levels of beneficial bioactive substances, such as polyphenols, ferulic acid, and gamma-aminobutyric acid. The polished rice (PR) had a high GI (83.10 ± 5.10). In contrast, these bioactive chemicals were retained or even improved during the parboiling and germination processes, resulting in rice forms with medium GI values.

### 6.1. Milling

Rice milling is a process that removes the husk and bran layers from paddy rice to produce edible white rice. It includes cleaning, dehusking/dehulling, polishing, and sorting steps. The production of brown rice does not require polishing. Brown rice has a bran layer and endosperm cell walls made of non-starch polysaccharides that act as a natural barrier, protecting the starchy endosperm from rapid digestion. However, the bran layer and germ/embryo are removed during polishing for white rice production, which significantly reduces the nutritional value of rice and increases the digestibility of rice starch. Studies consistently demonstrate that polished rice has a higher GI than less processed varieties, such as parboiled or unpolished brown rice [168,169]. By contrasting brown rice with white rice polished at different levels (2.4%, 4.4%, and 8.0%), Shobana et al. [159] looked at the impacts of polishing on rice in more detail. They discovered that polishing increases the number of accessible carbohydrates but drastically decreases nutrients, such as protein, fat, dietary fiber, γ-oryzanol, polyphenols, and antioxidant activity. According to Trinidad et al. [67] and Mohan et al. [170], eating brown rice causes blood sugar levels to rise more slowly and less dramatically. Evaluating the GI of an Indian rice variety (Bapatla–BPT-5204) under various polishing levels, Shobana et al. [159] provided additional confirmation of this. Undermilled rice (2.3% polishing) and white rice (9.7% polishing) had progressively higher GIs (73 and 79.6, respectively), whereas brown rice (0% polishing) had the lowest GI (57.6). Similarly, Sasaki et al. [171] found that while brown rice digests more slowly, white rice releases glucose more quickly. 

The degree of milling (DOM) is another factor that influences rice starch’s digestibility and glycemic index. Wang et al. [157] assessed Simiao rice milled at 0–12% levels, revealing that increased milling diminished SDS and RS, hence enhancing digestibility and eGI (77.98–85.55). The eGI had a negative correlation with fat, protein, dietary fiber, and resistant starch, while showing a positive correlation with amylose, illustrating the impact of milling on rice’s nutritional and digestibility characteristics. Furthermore, another study found that the DOM substantially affected the starch digestibility of cooked rice, as higher DOM decreases RDS and RS fractions while increasing the SDF fraction, providing insights for the creation of rice products with customized digestibility to satisfy healthier dietary requirements [172].

### 6.2. Extrusion

Rice extrusion is a food processing technique used to produce breakfast cereal, rice-based snacks, pasta, puffed rice, etc. [173]. Food products can be mixed, heated, extruded, and shaped using extrusion technology, which is a flexible and popular food processing technique [174]. According to screw design, the technology is divided into three primary categories: single, twin, and numerous screws. Due to its combination of high temperatures and strong shear forces, twin-screw extrusion is particularly effective at changing materials’ physical, chemical, and structural properties through friction and vibration [175]. Rice extrusion partially or completely disrupts the crystalline structure of starch, resulting in the development of amylose–lipid complexes, protein denaturation, and starch molecule fragmentation [176]. High-amylose rice cultivars are optimal for extrusion as they reduce cooking losses and promote the formation of amylose–lipid complexes, hence decelerating starch digestion. Extrusion is a versatile processing technique that can yield diverse products; however, fluctuations in raw material composition and processing circumstances may influence the quality of the end product. Consequently, it is important to meticulously oversee and refine production parameters while assessing the attributes of the extruded product to guarantee uniformity and excellence.

Extrusion processing of rice, whether conducted via hot or cold processes, yields a product with a reduced GI compared to milled rice or extruded wheat. Extrusion induces the gelatinization and retrogradation of starch, hence modifying its digestibility. The elevated temperatures and mechanical shear stresses during extrusion compromise the starch’s structure, facilitating the realignment of amylose molecules to create RS, which impedes digestion. According to research by Feng and Lee [177] and Zeng et al. [178], extruded rice has lower GI values and lower amounts of RDS. Fruit peels and other additives improve the nutritional profile of extruded rice by lowering the GI and increasing antioxidant activity. According to Ye et al. [179], the higher RS content of extruded texturized rice results in less starch hydrolysis, and three factors account for the decreased digestibility of extruded rice: (1) proteins are covalently cross-linked and therefore resistant to protease digestion, (2) proteins coat starch and prevent amylase action, and (3) the development of a complex structure in which proteins and starch entangle and are thus resistant to enzymatic breakdown. These processes work together such that extruded rice is digested more slowly and has a lower GI.

### 6.3. Baking

Baking effectively enhances the RS content in foods, decelerating starch digestion and improving the nutritional profile. The magnitude of this effect is contingent on the baking method employed. The baking time and temperature affect the structural transformations in starch, including gelatinization and retrogradation, which ultimately influence the digestibility of starch by enzymes. Effective management of these conditions is crucial for enhancing RS formation and optimizing the health benefits of baked goods. Giuberti et al. [180] conducted a study on gluten-free cookie recipes, substituting half of the commercial rice flour with RS ingredients. Among the recipes tested, cookies prepared with RS from debranched waxy rice starch exhibited the most notable decrease in GI, recording a value of 71. This underscores the potential of utilizing RS as a functional ingredient to develop healthier baked goods that exert a reduced effect on blood sugar levels. Another study focused on the formulation of low-GI gluten-free cookies utilizing high-amylose rice flour, achieved through the optimization of baking parameters (185 °C for 22 min) and the incorporation of carboxymethyl cellulose at a concentration of 0.8% [162]. Results showed that the RS content rose from 2.85% in flour to 7.20% in cookies, leading to a decrease in the predicted GI from 50.12 to 44.60 and the glycemic load from 30.07 to 17.51. Rakmai et al. [181] demonstrated that substituting rice flour with 10% resistant maltodextrin and 25% sucralose resulted in a reduction of the GI of pancakes to 51.9 for Jasmine and 49.3 for Sangyod, alongside a decrease in calories and carbohydrates and an increase in fiber and protein content. This underscores the potential of dietary fiber and low-calorie sweeteners for developing low-GI foods to manage non-communicable diseases. Despite the rising popularity of gluten-free, rice-based foods, comprehensive studies are needed to investigate the impact of variables, such as baking temperature, duration, and ingredient composition, on their starch digestibility, which may be important to individuals with dietary restrictions or metabolic disorders.

### 6.4. Cooking

Starchy crops are fundamental to numerous diets and are typically prepared through cooking before consumption. Cooking methods, such as boiling, steaming, frying, and pasteurization, enhance the bioavailability of nutrients, including starch, proteins, and antioxidants [182]. Starch granules absorb water and swell when heated in the presence of water, which is known as gelatinization. Upon cooking, starch granules are disrupted, releasing amylose and amylopectin, which are digested by enzymes, affecting the glycemic response [183].

The cooking process of rice induces starch gelatinization, which improves texture and increases the bioavailability of nutrients. The texture and nutritional properties of cooked rice are significantly influenced by the cooking time, temperature, and moisture content. Uncooked rice powder, with its lower gelatinized starch content, causes a slower rise in blood glucose levels after ingestion than cooked rice, which has more gelatinized starch [184]. Longer cooking times can result in increased starch swelling and splitting, which leads to a higher GI, as evidenced by Wolever et al. [185]. Cooking rice at elevated temperatures can increase starch leaching, resulting in a higher GI [186].

Cooking procedures substantially impact starch digestibility [6]. Elevated moisture content interferes with the crystalline structure of starch, facilitating gelatinization upon heating. This enhances the accessibility of starch to digestive enzymes, accelerating the degradation of glucose polymers and enhancing the rate of glucose release into the bloodstream [6,187]. Because of this, rice cooked with more moisture typically has a higher GI [188]. Insufficient moisture during cooking, on the other hand, prevents starch from expanding and gelatinizing and maintains its crystalline structure. By decreasing the starch’s accessibility to digestive enzymes, this lowers the GI and delays the release of glucose. Numerous studies have demonstrated that reduced moisture levels during rice preparation reduced starch digestion and aided in blood sugar stabilization [50,189,190]. Boiling is the conventional rice cooking method; however, pressure cooking offers a more rapid and energy-efficient alternative that enhances the digestibility of proteins and starches [191]. Furthermore, the storage and reheating of cooked rice influence the retrogradation process, modifying the starch structure and affecting its digestibility and texture [24]

Microwaving and stir-frying present convenient and diverse cooking options. For example, stir-frying freshly steamed rice lowers starch’s digestibility while increasing resistant starch (RS) levels. This occurs due to two primary mechanisms: (1) the production of amylose–lipid complexes, which are more difficult for digestive enzymes to break down, and (2) the lipid coating effect, which further inhibits starch hydrolysis [192]. Microwave heating was found to marginally lower rice’s GI compared to traditional cooking methods; however, the change was negligible [193].

More recent studies have revealed that the GI of rice could be decreased by optimizing cooking conditions, such as utilizing lower temperatures, shorter cooking times, and water-to-rice ratios, which produce a denser structure that prevents enzymatic breakdown [194]. Interestingly, conventional cooking produces a higher GI than retort cooking despite having a higher retrogradation enthalpy, because retort cooking raises the resistant starch (RS) content due to high heat and pressure [195]. Wang et al. illustrated that particular cooking methods, including high-temperature soaking, brief heating, and rapid cooling, markedly decreased starch digestibility in pigmented brown rice due to the enhanced density of molecular chain packing, which was validated by FTIR and SAXS analyses [196]. The structural alteration restricted enzyme accessibility and resulted in elevated SDS and RS levels.

### 6.5. Parboiling

Parboiling is a process of partially cooking rice in its husk. It is designed to improve rice’s cooking and nutritional qualities. Parboiled rice is produced by soaking, steaming, draining, and drying the paddy rice. Parboiling started in India, and it is chiefly practiced in many other Asian countries and sub-Saharan African countries [197]. Parboiled rice accounts for approximately 20% of the world’s net grain processed [198]. During the parboiling process, the starch granules become irreversibly attached, thus changing them from a crystalline to an amorphous form [199]. Gelatinization of the starch granules results in the production of parboiled rice, which affords benefits to the grain, such as an improved yield (lower number of broken grains), sterilization, inhibited enzyme activities, resistance to insect attack, increased shelf life, and preservation of vitamins and minerals.

Parboiling has been demonstrated to reduce the GI of rice, with a study claiming an average GI of 53.9 [200]. Rondanelli et al. examined Italian Indica and Japonica parboiled rice, assessing the glycemic index of different types and processing techniques. Results indicated that most parboiled rice varieties had low glycemic index values, except for red rice (medium GI) and rice cakes (high GI). As a result, parboiled rice is a better option than non-parboiled rice for controlling blood sugar levels [201]. In healthy people, parboiled rice has a reduced glycemic response compared to non-parboiled rice [202]. This is explained by the reduction in starch digestibility caused by protein–starch interactions, higher protein concentration, and starch retrogradation. A review suggests that better glycemic control might result from the higher levels of calcium, selenium, and vitamin B6 found in parboiled rice compared to white rice, brown rice, and pasta [203]. Selenium lowers oxidative stress, linked to diabetes, calcium helps insulin function, and reduced levels of phytic acid enhance calcium absorption. These elements combine to make parboiled rice more healthful for controlling blood sugar levels.

Moreover, parboiled rice has less phytic acid than brown rice. Reduced amounts of phytic acid in parboiled rice may increase calcium bioavailability and promote better metabolic health because it inhibits calcium absorption and other minerals [203].

### 6.6. Retrogradation

Starch undergoes gelatinization upon heating in water, which disrupts its granular structure and induces an order-to-disorder shift [204]. Retrogradation occurs when cooked starch is cooled and stored for a period of time. It causes the disordered amylose and amylopectin chains to re-associate into a newly ordered structure, which changes the characteristics of starch, such as digestibility, flavor, texture, and viscosity [205].

The time and temperature of storage play essential roles in starch retrogradation [205]. Retrogradation has been demonstrated to change the RS content of starch, affecting its digestibility. For example, research using waxy rice starch found that retrogradation initially resulted in a drop in RS content. However, when the starch was held at 4 °C for more extended periods (2–7 days), the RS% rose over time [206]. This shows that storage time is an essential factor in determining the level of retrogradation and its impact on RS development.

The differential scanning calorimetry (DSC) study of retrograded rice gels stored for 1, 3, and 6 days revealed single endothermic peaks ranging from 35.5 to 69.0 °C, with differing enthalpies signifying starch recrystallization. Retrograded gels had lower peak temperatures than gelatinized starch, indicating structural alterations when starch chains reassemble into a more organized, crystalline configuration [207]. Similarly, another study examined the impact of storage duration and temperature on retrograded rice starch across five indigenous varieties. DSC results revealed that keeping cooked rice at −20 °C for 12 h optimized the degree of retrogradation (DR%) and RS% while reducing non-resistant starch (NRS%). Extended storage durations and reduced temperatures elevated the gelatinization temperature (Tp) and enhanced textural characteristics, revealing a negative correlation between Tp and RS%. Retrograded starch at lower temperatures for extended periods increases the resistant starch percentage, lowers the glycemic index, and enhances functionality, rendering it suitable for formulating healthier food products [208].

## 7. Conclusions and Future Recommendations

This review has illustrated that the amylose and amylopectin ratios, granule shapes, and glycemic indices of rice vary with the rice variety, degree of milling, and processing and cooking techniques. Rice with higher amylose content, a low degree of milling, and high anthocyanin content (colored rice) exhibited lower GI but reduced functional properties that affect their palatability, while the glutinous rice and white rice have better eating quality but a much higher GI, which is undesirable for diabetic and obese populations. Optimization of processing conditions can help to reduce the GI and balance the nutritional value and palatability of rice. For example, a moderate degree of milling, methods promoting starch retrogradation, and minimizing starch damage and gelatinization could significantly reduce its digestibility and alter its texture. Moreover, a reduction of the digestibility and GI of rice can also be achieved by increasing the complexation between starch and other constituents, particularly dietary fiber and polyphenol-rich ingredients. However, dietary fiber and polyphenol also interact with proteins and lipids to form complexes with reduced digestibility. In addition, dietary fiber and polyphenols may also influence the absorption of micronutrients, such as minerals and vitamins. Therefore, care must be taken when formulating rice-mix or rice-based products with dietary fiber and polyphenol-rich ingredients to avoid overdose. Comprehending these characteristics is crucial for enhancing rice-based diets to tackle health issues, including glycemic regulation and nutrient assimilation, while maintaining cultural and dietary importance.

Future research should concentrate on creating low-glycemic rice varieties through sustainable and environmentally friendly green technologies to satisfy the increasing need for healthier carbohydrate sources. Enhancing processing procedures, including parboiling, milling, extrusion, and cooking, is essential to reduce nutrient loss and improve the functional characteristics of rice starch. Furthermore, additional research is required to elucidate the intricate interactions between rice starch and other food constituents, including proteins, lipids, dietary fiber, and polyphenols, to formulate functional diets with improved health advantages and to minimize the undesirable effects. To better understand the starch–polyphenols interaction, there is a need for more comparative studies under standardized conditions. Further studies are needed on the impacts of novel cooking techniques, including infrared radiation heating, pressure cooking, and microwave heating, on rice starch digestibility and nutrient preservation, which may yield valuable insights for consumers and the food sector.

## Figures and Tables

**Table 2:** Effects of food ingredient interaction on rice starch physicochemical properties, digestibility, and GI.

Interaction Type	Rice Starch Type	Types of Constituents	Mechanism	Study Results	Reference
Starch–Protein Interaction	Waxy rice	Whey protein isolate (WPI), soy protein isolate (SPI), pea protein isolate (PPI)	Starch–protein interaction through hydrogen bonding and hydrophobic interactions limited water mobility	Reduced RDS and increased RS content; low glycemic index	[102]
	Polished long-grain Jasmine	Albumin, globulin, glutelin, prolamin, and acetic rice proteins	Protein forms a protective network around starch granules, reducing their accessibility to digestive enzymes	A negative correlation was observed between residual rice protein and digestibility	[103]
	Japonica	Endogenous/exogenous rice protein hydrolysate mixtures	Enzyme activity is inhibited by peptides or free amino acids	Raw rice starch’s digestion rate constants (k) dropped from 2.34 × 10^−2^ to 2.16 × 10^−2^ and 2.09 × 10^−2^ min^−1^, whereas cooked rice starch’s k dropped from 4.28 × 10^−2^ to 3.24 × 10^−2^ and 2.94 × 10^−2^ min^−1^, with a significantly lower GI	[104]
	Native long Indica RF—rice flour	Endogenous proteins RF-P: rice flour protein	Endogenous proteins encase starch granules, limiting their expansion and interaction with digestive enzymes	Based on the first model kinetics, RF > RF-P showed increasing trends in C∞, HI, and eGI values	[105]
	Japonica	Rice protein and amino acids	Amino acids inhibit rice starch digestion by reducing enzymes’ access to starch and enhancing the stability of starch granules, creating a more compact structure	A complex formed between rice starch and amino acids, resulting in increased SDS and RS content and the development of low-GI-based foods	[106]
	Starch	Rice protein hydrolysate	Native and pepsin hydrolyzed proteins enhanced starch retrogradation, whereas pepsin–pancreatin hydrolyzed protein decreased retrogradation but enhanced V-type complexes and inhibited α-amylase	Protein hydrolysates increased in SDS and RS content	[107]
Starch–Lipid Interaction	Low/medium/high GI rice varieties	Cooking oils and ghee	The addition of fat or oil delays gastric emptying, enhances incretin secretion, and forms amylose–lipid complexes that decrease starch digestion by α-amylase	Combining rice with ghee and soybean oil significantly lowers the GI values of Shaktiman, Hue, and Kalashree rice varieties by 3.83%, 24.90%, and 7.96% for ghee and 3.65%, 24.12%, and 7.61% for soybean oil	[108]
	High-lipid mutants (ALK3 and RS4)	Rice endosperm lipids	Pancreatic lipase hydrolysis of triglycerides makes the lipid–starch complex vulnerable to α-amylase breakdown	High-lipid white rice holds significant promise for creating functional rice-based foods, offering a comparatively lower GI and higher γ-oryzanol levels	[109]
	Seven rice mutants with different RS contents	Native rice lipids	Starch–lipid complexes and short chains (DP 8–12) significantly affect starch digestion, while the integrity of aggregated starch and the morphology of spherical starch granules may also influence digestibility	There was a positive correlation between lipid content RS content and a negative correlation between lipid content and eGI	[110]
	Sao Hai rice (SH)	Coconut oil and rice bran oil at 2.5, 5, and 7.5% (*w*/*w*, based on uncooked rice)	The formation of the amylose–lipid complex increases crystallinity, which exhibits a V-type crystalline structure	Rice bran oil at 2.5% resulted in cooked rice with the highest SDS and RS and the lowest eGI	[111]
	Rice cooked with FiberCreme	Coconut oil made with FiberCreme	The interaction between rice starch and lipid and fiber in FiberCreme led to increased RS	Notable reduction in the GI, decreasing from 79.8 to 70.7	[112]
	Rice starch and rice flour	Coconut oil (CO), rice bran oil (RO), palm oil (PO), and soybean oil (SO)	Adding CO and PO improved the melting temperatures, dissociation enthalpies, and V-type crystalline order of the amylose–lipid complex compared to RO and SO	High levels of saturated fatty acids in CO and PO decreased RDS and increased RS in cooked rice starch and flour	[113]
	Starchy foods	Glycerol monostearate (GMS)/stearic acid (SA)	Thermal shear force in HE-3DP facilitated hydrophobic interactions, resulting in a compact V-type starch–lipid complex that exhibits enhanced enzymatic resistance	The content of slowly digestible starch (SDS) and resistant starch (RS) attained 25.06% with an added SA content of 10%	[114]
Starch–Dietary Fiber Interaction	High-fiber white rice (HFWR)	Dietary fiber (fivefold higher than white rice)	A high dietary fiber content, along with the presence of amylose tightly bound glucose chains, makes amylose less available for amylolytic attack compared to the amylopectin	HFWR exhibited a medium GI, which is 23% lower than that of commercial white rice	[115]
	Pigmented and non-pigmented rice	Dietary fiber	Dietary fibers, including soluble/insoluble fiber, and resistant starch affect the morphology, digestibility, and antioxidant activity cooked grain in the gut	GI is negatively correlated with levels of RS and SDS but positively correlated with RDS	[116]
	Extruded rice grain	Soybean dietary fiber (DF)	Influences the molecular interactions, microstructure, and pasting properties	GI decreased when the DF content exceeded 6%; lower RDS and higher RS	[117]
	White rice	FiberCreme (isomalto oligosaccharides, inulin, iso maltodextrin)	Modify the starch structure by weakening hydrogen bonds, reducing enzyme binding sites, and producing a viscous gel matrix with dense molecular network that limits surface contact and slows enzyme penetration	Increased the RS content from 3.40 to 5.21%, decreasing the GI	[112]
Starch–Polyphenol Interaction	Various kinds of starch	(+)Catechin, EGCG, quercetin, kaempferol, naringenin, hesperidin, trans-ferulic acid, and p-coumaric acid	(+)Catechin, epigallocatechin gallate, is the most effective starch digestion inhibitor due to its abundant hydroxyl groups, galloyl moiety, and lack of -OCH_3_ and glycosylation, which improve binding and α-amylase inhibition	(+)Catechin, epigallocatechin gallate (20 mg) significantly reduced RDS, TS, and eGI, regardless of the starch type or the timing of addition	[118]
	Rice (Basmati rice)	Cinnamon and turmeric	The bioactive compounds in cinnamon and turmeric alter the enzyme’s structure through non-covalent interactions, such as hydrogen bonding and hydrophobic interactions, ultimately reducing catalytic activity	Reduced GI level of rice from 66.6 to 46.0 and glycemic load (GL) from 33.0 to 23.0	[119]
	Cr-fortified parboiled rice (Cr-PR)	Coated with 3%, 6%, and 9% herbal extract from cinnamon, pandan, bay leaf	Formed starch–polyphenol complexes through hydrogen bonding, delaying starch digestion and glucose absorption	Cr-PR coated with a 3% herbal extract had higher RS levels than those with 6% and 9% herbal extracts; however, the lowest GI (29–30) was achieved by the Cr-PR coated with 6–9% cinnamon extract	[120]

**Table 3:** Effects of rice processing on the composition, structure, functional properties, and glycemic index of rice starches.

Processing Method	Processing Parameters	Possible Effects	Reference
Milling	Degree of milling (DOM): 2%, 4%, 6%, 8%, 10%, and 12%	Increased DOMs lowered SDS and RS content and increased rice digestibility and eGI	[157]
	Semi-dry-milled (SRF), wet-milled (WRF), dry-milled (DRF), jet-milled (JRF)	WRF and SRF had substantially lower eGI values than DRF and JRF	[158]
	Polishing to a degree of 2.3% and 9.7% based on the amount of bran removed	Increasing degree of milling leads to higher glycemic responses	[159]
Polishing	Producing white rice from brown rice by removing bran and germs	Polishing removed bran (DF, vitamins, minerals), germs (protein, lipid) and bioactives; polishing increased the GI from 55.10 ± 5.37 to 83.10 ± 5.10 and reduced the nutritional value of rice	[156]
Extrusion	Twin-screw extruder with co-rotating screw configurations and a 6 mm die; a three-section heating procedure (75–85 °C, 105–115 °C, and 80–90 °C)	Extrusion cooking resulted in a rough surface and converted the crystalline structure from an A-type to a mixture of B- and V-types; lowered the equilibrium starch hydrolysis rate (*C_∞_*) and kinetic constant (*k*) of broken rice	[160]
	Barrel length relative to barrel diameter is 25:1, with maximum single-shaft torque at 52.5 Nm, temperature at 400 °C, pressure at 000 psi, and screw speed at 500 rpm	Chemically modified rice starch through one-step reactive extrusion (REX) demonstrates that esterification retards starch breakdown and cross-linking accelerates and increases the RS content and the reduced (pGI) score compared to native rice	[161]
Baking	Rice cookie baking temperature, 170–190 °C; baking time, 12–25 min; and 0.2–1.0% of carboxymethyl cellulose	Optimal conditions for gluten-free, low-GI cookies from rice flour are 185 °C for 22 min with 0.8% carboxymethyl cellulose. RS increased from 2.85% in rice flour to 7.20% in cookies, while pGI and glycemic load decreased from 50.12–30.07 to 44.60–17.51	[162]
Parboiling	CR = cooked rice, FR = fermented rice, PR = parboiled rice, PCR = parboiled cooked rice, PFR = parboiled fermented rice	The PCR had the lowest pGI and the highest RS (2.7%), while raw fermented rice had the highest pGI and the lowest RS (0.31%)	[163]
	PBR (parboiled rice): soaked at 30–40 °C for 6–8 h, steamed 8–12 min, and then dried in an oven at 50–60 °C to a moisture content of 14%. GPBR (germinated parboiled rice): soaked at 30–40 °C for 12–15 h, changed water after 3–4 h, steamed for 8–12 min, and dried at 50–60 °C to 14% moisture content	PBR and GPBR exhibited higher polyphenol and γ-aminobutyric acid (GABA) contents but a lower GI than brown rice and parboiled white rice, indicating that parboiling and germination delayed carbohydrate digestion and glucose absorption	[156]
	Autoclave at 105 °C, 15 psi for 12 min, soak at 32 ± 2 °C, and then autoclave for 8 min. Cool and dry at 40 °C to achieve 13 ± 1% moisture content	Reduced in vitro starch digestibility, projected glycemic index (pGI), and protein digestibility by approximately 1.14-fold	[164]
Cooking	Boiling, dry heating, steaming, rinsing, and steam cooking	Pre-treated and cooked grains increased the RS content (5–20%) and decreased the eGI	[165]
	Steaming levels: 30, 35, and 40 min	Steaming duration influences amylose and amylopectin levels and also reduced the GI (67.24 ± 0.02 to 50.41 ± 0.23 g)	[166]
	Natural high-resistant starch (RS) rice varieties roasted at 150 °C for 30 to 50 min at different moisture levels	Improved the thermal stability of the rice, shortened the chain length distribution of amylopectin of rice starch, increased the RS content, resulted in reduced digestibility, generated desirable flavoring compounds, and reduced water solubility and swelling potential	[167]

## Data Availability

The original contributions presented in the study are included in the article; further inquiries can be directed to the corresponding author.

## References

[1] Chandio A.A., Gokmenoglu K.K., Ahmad M., Jiang Y. (2022). Towards sustainable rice production in Asia: The role of climatic factors. Earth Syst. Environ..

[2] United States Department of Agriculture (2024). World agricultural production: Ju Foreign Agricultural Service. https://www.fas.usda.gov/data/production/commodity/0422110.

[3] Al-Hashimi A.M. (2023). A review: Growing rice in the controlled environments. Biosci. Biotechnol. Res. Asia.

[4] Kumar A., Sahoo U., Lal M.K., Tiwari R.K., Lenka S.K., Singh N.R., Gupta O.P., Sah R.P., Sharma S. (2022). Biochemical markers for low glycemic index and approaches to alter starch digestibility in rice. J. Cereal Sci..

[5] Lovegrove A., Kosik O., Bandonill E., Abilgos-Ramos R., Romero M., Sreenivasulu N., Shewry P. (2019). Improving rice dietary fibre content and composition for human health. J. Nutr. Sci. Vitaminol..

[6] Reed M.O., Ai Y., Leutcher J.L., Jane J.L. (2013). Effects of cooking methods and starch structures on starch hy-drolysis rates of rice. J. Food Sci..

[7] Chiu Y.-T., Stewart M.L. (2013). Effect of variety and cooking method on resistant starch content of white rice and subsequent postprandial glucose response and appetite in humans. Asia Pac. J. Clin. Nutr..

[8] Dwiningsih Y., Alkahtani J. (2023). Glycemic index of diverse rice genotypes and rice products associated with health and diseases. ASSET.

[9] Howlader M.Z.H., Biswas S.K. (2009). Screening for nutritionally rich and low glycemic index Bangladeshi rice varieties. Final Rep. CF.

[10] Van Ngo T., Kunyanee K., Luangsakul N. (2023). Insights into recent updates on factors and technologies that modulate the glycemic index of rice and its products. Foods.

[11] Srikaeo K. (2023). Application of a rapid in vitro method based on glucometer for determination of starch digestibility and estimated glycemic index in rice. Starch-Starke.

[12] Yao F., Li C., Li J., Chang G., Wang Y., Campardelli R., Perego P., Cai C. (2023). Effects of different cooking methods on glycemic index, physicochemical indexes, and digestive characteristics of two kinds of rice. Processes.

[13] Fitzgerald M.A., Rahman S., Resurreccion A.P., Concepcion J., Daygon V.D., Dipti S.S., Kabir K.A., Klingner B., Morell M.K., Bird A.R. (2011). Identification of a major genetic determinant of glycaemic index in rice. Rice.

[14] Maji B. (2019). Introduction to natural polysaccharides. Functional Polysaccharides for Biomedical Applications.

[15] Fernandes J.-M., Madalena D.A., Pinheiro A.C., Vicente A.A. (2020). Rice in vitro digestion: Application of INFOGEST harmonized protocol for glycemic index determination and starch morphological study. J. Food Sci. Technol..

[16] Trinh K.S. (2015). Recrystallization of starches by hydrothermal treatment: Digestibility, structural, and physicochemical properties. J. Food Sci. Technol..

[17] Huang M., Hu L., Chen J., Cao F. (2022). In vitro testing indicates an accelerated rate of digestion of starch into glucose of cooked rice with the development of low amylose rice in China. Food Chem. X.

[18] Zaman S.A., Sarbini S.R. (2016). The potential of resistant starch as a prebiotic. Crit. Rev. Biotechnol..

[19] Englyst H.N., Kingman S.M., Cummings J.H. (1992). Classification and measurement of nutritionally important starch fractions. Eur. J. Clin. Nutr..

[20] Sajilata M.G., Singhal R.S., Kulkarni P.R. (2006). Resistant starch—A review. Compr. Rev. Food Sci. Food Saf..

[21] Juliano B.O., Tuaño A.P.P., Bao J. (2019). 2-Gross structure and composition of the rice grain. Rice.

[22] Wang L., Gong Y., Li Y., Tian Y. (2020). Structure and properties of soft rice starch. Int. J. Biol. Macromol..

[23] Fitzgerald M.A., McCouch S.R., Hall R.D. (2009). Not just a grain of rice: The quest for quality. Trends Plant Sci..

[24] Boers H.M., Hoorn J.S.T., Mela D.J. (2015). A systematic review of the influence of rice characteristics and processing methods on postprandial glycaemic and insulinaemic responses. Br. J. Nutr..

[25] Zhuang J., Li C. (2025). Addition of rice protein inhibits rice starch digestibility by enlarging the hydrogel pore size and promoting the formation of resistant starch with a DP around 150. Food Hydrocoll..

[26] Wang Y., Ral J.-P., Saulnier L., Kansou K. (2022). How Does Starch Structure Impact Amylolysis? Review of Current Strategies for Starch Digestibility Study. Foods.

[27] Devi K.J., Semmichon S., Jarh A., Sinha M., Gogoi M. (2024). Factors affecting starch digestibility and glycemic index of rice: A comprehensive review. Plant Arch..

[28] Guzman M. (2016). Rice With a Hawaiian Touch. J. Ren. Nutr..

[29] Priya T.S.R., Nelson A.R.L.E., Ravichandran K., Antony U. (2019). Nutritional and functional properties of coloured rice varieties of South India: A review. J. Ethn. Foods.

[30] Srichuwong S., Curti D., Austin S., King R., Lamothe L., Gloria-Hernandez H. (2017). Physicochemical properties and starch digestibility of whole grain sorghums, millet, quinoa and amaranth flours, as affected by starch and non-starch constituents. Food Chem..

[31] Rondanelli M., Ferrario R.A., Barrile G.C., Guido D., Gasparri C., Ferraris C., Cavioni A., Mansueto F., Mazzola G., Patelli Z. (2023). The Glycemic Index of Indica and Japonica Subspecies Parboiled Rice Grown in Italy and the Effect on Glycemic Index of Different Parboiling Processes. J. Med. Food.

[32] Gunaratne A., Wu K., Kong X., Gan R., Sui Z., Kumara K., Ratnayake U.K., Senarathne K., Kasapis S., Corke H. (2019). Physicochemical properties, digestibility and expected glycaemic index of high amylose rice differing in length-width ratio in Sri Lanka. Int. J. Food Sci. Technol..

[33] Afandi F. (2023). The Correlation Between Amylopectin Chain-Length and Glycemic Index Value of Carbohydrate Foods: A Review. Food Sci. J. Food Sci. Technol..

[34] Atkinson F.S., Foster-Powell K., Brand-Miller J.C. (2008). International tables of glycemic index and glycemic load values: 2008. Diabetes Care.

[35] Juliano B.O. (1993). Rice in human nutrition. Int. Rice Res. Inst..

[36] Frei M., Becker K. (2004). On Rice, Biodiversity & Nutrients. University of Hohenheim, Stuttgart. https://citeseerx.ist.psu.edu/document?repid=rep1&type=pdf&doi=d9dd183bb8763f44ad34be8906d60ba5f0b5383b.

[37] Ghasemzadeh A., Karbalaii M.T., Jaafar H.Z., Rahmat A. (2018). Phytochemical constituents, antioxidant activity, and antiproliferative properties of black, red, and brown rice bran. Chem. Cent. J..

[38] Somaratne G., Prasantha B., Dunuwila G., Chandrasekara A., Wijesinghe D., Gunasekara D. (2017). Effect of polishing on glycemic index and antioxidant properties of red and white basmati rice. Food Chem..

[39] Hu Z., Tang X., Zhang M., Hu X., Yu C., Zhu Z., Shao Y. (2018). Effects of different extrusion temperatures on extrusion behavior, phenolic acids, antioxidant activity, anthocyanins and phytosterols of black rice. RSC Adv..

[40] Zhang M.W., Zhang R.F., Zhang F.X., Liu R.H. (2010). Phenolic profiles and antioxidant activity of black rice bran of different commercially available varieties. J. Agric. Food Chem..

[41] Sun Q., Spiegelman D., van Dam R.M., Holmes M.D., Malik V.S., Willett W.C., Hu F.B. (2010). White rice, brown rice, and risk of type 2 diabetes in US men and women. Arch. Intern. Med..

[42] Paiva F.F., Vanier N.L., Berrios J.D.J., Pinto V.Z., Wood D., Williams T., Pan J., Elias M.C. (2016). Polishing and parboiling effect on the nutritional and technological properties of pigmented rice. Food Chem..

[43] Hu E.A., Pan A., Malik V., Sun Q. (2012). White rice consumption and risk of type 2 diabetes: Meta-analysis and systematic review. BMJ.

[44] Kumari S.D.P., Devi G.N., Chamundeswari N. (2021). Glycemic index studies in rice (*Oryza sativa* L.) varieties developed by ANGRAU. Int. J. Agric. Sci..

[45] Subramaniam J., Yusof B.N.M., Ngan H.B., Ismail A., Azlan A. (2014). Relationship between Amylose Con-tent and Glycemic Index of Commonly Consumed White Rice. IOSR J. Agric. Vet. Sci..

[46] Setyaningsih W., Hidayah N., Saputro I.E., Lovillo M.P., Barroso C.G. Study of glutinous and non-glutinous rice (*Oryza sativa*) varieties on their antioxidant compounds. Proceedings of the International Conference on Plant, Marine and Environmental Sciences.

[47] Guo L., Zhang J., Hu J., Li X., Du X. (2015). Susceptibility of glutinous rice starch to digestive enzymes. Carbohydr. Polym..

[48] Atkinson F.S., Brand-Miller J.C., Foster-Powell K., Buyken A.E., Goletzke J. (2021). International tables of glycemic index and glycemic load values 2021: A systematic review. Am. J. Clin. Nutr..

[49] Nanri A., Mizoue T., Noda M., Takahashi Y., Kato M., Inoue M., Tsugane S. (2010). Rice intake and type 2 diabetes in Japanese men and women: The Japan Public Health Center–based Prospective Study. Am. J. Clin. Nutr..

[50] Kaur B., Ranawana V., Henry J. (2013). The glycemic index of rice and rice products: A review, and table of GI values. Crit. Rev. Food Sci. Nutr..

[51] Sheng T., Mang L., Wu Y., Zhu H., Ha C., Xiao S., Yu Z., Zhou Y. (2025). Functions of high glycemic index carbohydrates: Exploring the effect of amorphous rice starch digestibility on glycometabolism. Int. J. Biol. Macromol..

[52] Tuaño A.P.P., Barcellano E.C.G., Rodriguez M.S. (2021). Resistant starch levels and in vitro starch digestibility of selected cooked Philippine brown and milled rices varying in apparent amylose content and glycemic index. Food Chem. Mol. Sci..

[53] Wiruch P., Naruenartwongsakul S., Chalermchart Y. (2019). Textural properties, resistant starch, and in vitro starch digestibility as affected by parboiling of brown glutinous rice in a retort pouch. Curr. Res. Nutr. Food Sci. J..

[54] Sen S., Chakraborty R., Kalita P. (2020). Rice-not just a staple food: A comprehensive review on its phytochemicals and therapeutic potential. Trends Food Sci. Technol..

[55] Saleh A.S., Wang P., Wang N., Yang L., Xiao Z. (2019). Brown rice versus white rice: Nutritional quality, po-tential health benefits, development of food products, and preservation technologies. Compr. Rev. Food Sci. Food Saf..

[56] Thondre P., Monro J., Mishra S., Henry C. (2010). High molecular weight barley β-glucan decreases particle breakdown in chapattis (Indian flat breads) during in vitro digestion. Food Res. Int..

[57] Zhang B., Qiao D., Zhao S., Lin Q., Wang J., Xie F. (2021). Starch-based food matrices containing protein: Re-cent understanding of morphology, structure, and properties. Trends Food Sci. Technol..

[58] Sapwarobol S., Saphyakhajorn W., Astina J. (2021). Biological Functions and Activities of Rice Bran as a Functional Ingredient: A Review. Nutr. Metab. Insights.

[59] Chai Y., Wang M., Zhang G. (2013). Interaction between amylose and tea polyphenols modulates the postprandial glycemic response to high-amylose maize starch. J. Agric. Food Chem..

[60] Rattanamechaiskul C., Soponronnarit S., Prachayawarakorn S., Tungtrakul P. (2013). Optimal operating conditions to produce nutritious partially parboiled brown rice in a humidified hot air fluidized bed dryer. Dry. Technol..

[61] Wang H., Zhu S., Ramaswamy H.S., Du Y., Yu Y., Wu J. (2021). Dynamics of texture change and in vitro starch digestibility with high-pressure, freeze-thaw cycle, and germination-parboiling treatments of brown rice. Trans. ASABE.

[62] Wu J., McClements D.J., Chen J., Hu X., Liu C. (2016). Improvement in nutritional attributes of rice using su-perheated steam processing. J. Funct. Foods.

[63] Kim J.Y., Kim J.H., Kim S.H., Lee S.S. (2008). Meal replacement with mixed rice is more effective than white rice in weight control, while improving antioxidant enzyme activity in obese women. Nutr. Res..

[64] Meera K., Smita M., Haripriya S., Sen S. (2019). Varietal influence on antioxidant properties and glycemic index of pigmented and non-pigmented rice. J. Cereal Sci..

[65] Zhu R., Fan Z., Han Y., Li S., Li G., Wang L., Ye T., Zhao W. (2019). Acute effects of three cooked non-cereal starchy foods on postprandial glycemic responses and in vitro carbohydrate digestion in comparison with whole grains: A randomized trial. Nutrients.

[66] Abubakar B., Zawawi N., Omar A.R., Ismail M. (2017). Predisposition to insulin resistance and obesity due to staple consumption of rice: Amylose content versus germination status. PLoS ONE.

[67] Trinidad T.P., Mallillin A.C., Encabo R.R., Sagum R.S., Felix A.D., Juliano B.O. (2013). The effect of apparent amylose content and dietary fibre on the glycemic response of different varieties of cooked milled and brown rice. Int. J. Food Sci. Nutr..

[68] Panlasigui L.N., Thompson L.U. (2006). Blood glucose lowering effects of brown rice in normal and diabetic subjects. Int. J. Food Sci. Nutr..

[69] Kumar A., Sahu C., A Panda P., Biswal M., Sah R.P., Lal M.K., Baig M.J., Swain P., Behera L., Chattopadhyay K. (2020). Phytic acid content may affect starch digestibility and glycemic index value of rice (*Oryza sativa* L.). J. Sci. Food Agric..

[70] Nyambe-Silavwe H., Villa-Rodriguez J.A., Ifie I., Holmes M., Aydin E., Jensen J.M., Williamson G. (2015). Inhibition of human α-amylase by dietary polyphenols. J. Funct. Foods.

[71] Drechsler K.C., Bornhorst G.M. (2018). Modeling the softening of carbohydrate-based foods during simulated gastric digestion. J. Food Eng..

[72] Farooq M.A., Murtaza M.A., Aadil R.M., Arshad R., Rahaman A., Siddique R., Hassan S., Akhtar H.M.S., Manzoor M.F., Karrar E. (2021). Investigating the structural properties and in vitro digestion of rice flours. Food Sci. Nutr..

[73] Klunklin W., Savage G. (2018). Physicochemical, antioxidant properties and in vitro digestibility of wheat–purple rice flour mixtures. Int. J. Food Sci. Technol..

[74] Farooq A.M., Li C., Chen S., Fu X., Zhang B., Huang Q. (2018). Particle size affects structural and in vitro digestion properties of cooked rice flours. Int. J. Biol. Macromol..

[75] Zhang M., Guo B., Zhang R., Chi J., Wei Z., Xu Z., Zhang Y., Tang X. (2006). Separation, pu-rification and identification of antioxidant compositions in black rice. Agric. Sci. China.

[76] Sangma H.C.R., Parameshwari S. (2021). Health benefits of black rice (*Zizania aqatica*)—A review. Mater. Today: Proc..

[77] Ito V.C., Lacerda L.G. (2019). Black rice (*Oryza sativa* L.): A review of its historical aspects, chemical composi-tion, nutritional and functional properties, and applications and processing technologies. Food Chem..

[78] Shen Y., Jin L., Xiao P., Lu Y., Bao J. (2009). Total phenolics, flavonoids, antioxidant capacity in rice grain and their relations to grain color, size and weight. J. Cereal Sci..

[79] Zhang H., Kai G., Xia Y., Wang G., Ai L. (2020). Antioxidant and in vitro digestion property of black rice (*Oryza sativa* L.): A comparison study between whole grain and rice bran. Int. J. Food Eng..

[80] An J.S., Bae I.Y., Han S.-I., Lee S.-J., Lee H.G. (2016). In vitro potential of phenolic phytochemicals from black rice on starch digestibility and rheological behaviors. J. Cereal Sci..

[81] Hou Z., Qin P., Zhang Y., Cui S., Ren G. (2013). Identification of anthocyanins isolated from black rice (*Oryza sativa* L.) and their degradation kinetics. Food Res. Int..

[82] Alves G.H., Ferreira C.D., Vivian P.G., Monks J.L.F., Elias M.C., Vanier N.L., de Oliveira M. (2016). The revisited levels of free and bound phenolics in rice: Effects of the extraction procedure. Food Chem..

[83] Aalim H., Wang D., Luo Z. (2021). Black rice (*Oryza sativa* L.) processing: Evaluation of physicochemical properties, in vitro starch digestibility, and phenolic functions linked to type 2 diabetes. Food Res. Int..

[84] Shahidi F., Danielski R., Rhein S.O., Meisel L.A., Fuentes J., Speisky H., Schwember A.R., de Camargo A.C. (2022). Wheat and rice beyond phenolic acids: Genetics, identification database, antioxidant properties, and potential health effects. Plants.

[85] Xie F., Lei Y., Han X., Zhao Y., Zhang S. (2020). Antioxidant ability of polyphenols from black rice, buckwheat and oats: In vitro and in vivo. Czech J. Food Sci..

[86] Goufo P., Trindade H. (2014). Rice antioxidants: Phenolic acids, flavonoids, anthocyanins, proanthocyanidins, tocopherols, tocotrienols, *γ*-oryzanol, and phytic acid. Food Sci. Nutr..

[87] Tai L., Huang S., Zhao Z., Huang G. (2021). Chemical composition analysis and antioxidant activity of black rice pigment. Chem. Biol. Drug Des..

[88] Li X., Chen W., Gao J., Gao W., Zhang Y., Zeng H., Zheng B. (2023). Structural changes of butyrylated lotus seed starch and its impact on the gut microbiota of rat in vitro fermentation. Food Hydrocoll..

[89] Ou S.J.L., Fu A.S., Liu M.H. (2023). Impact of starch-rich food matrices on black rice anthocyanin accessibility and carbohydrate digestibility. Foods.

[90] Yawadio R., Tanimori S., Morita N. (2007). Identification of phenolic compounds isolated from pigmented rices and their aldose reductase inhibitory activities. Food Chem..

[91] Zeng Y.-W., Yang J.-Z., Pu X.-Y., Du J., Yang T., Yang S.-M., Zhu W.-H. (2013). Strategies of functional food for cancer prevention in human beings. Asian Pac. J. Cancer Prev..

[92] Sompong R., Siebenhandl-Ehn S., Linsberger-Martin G., Berghofer E. (2011). Physicochemical and antioxida-tive properties of red and black rice varieties from Thailand, China and Sri Lanka. Food Chem..

[93] Hettiarachchi H., Ribeira S., Prasantha B., Wickramasinghe H. (2016). Diversity of physical and cooking quality characters of selected traditional and improved rice varieties in Sri Lanka. Sri Lankan J. Biol..

[94] Abeysekera W., Somasiri H., Premakumara G., Bentota A., Rajapakse D., Ediriweera N. (2008). Cooking and eating quality traits of some Sri Lankan traditional rice varieties across Yala and Maha seasons. Trop. Agric. Res..

[95] Prasantha B.D.R. (2018). Glycemic index of four traditional red pigmented rice. Integr. Food Nutr. Metab..

[96] Wu M., Wu C., Wang Y., Bian X., Liang D., Zhang G., Liu X., Zhang N. (2025). Correlation between gastrointestinal index (GI) and the structure and physicochemical properties of rice starch from different varieties and colors. Carbohydr. Polym. Technol. Appl..

[97] Zhang W., Zhu H., Rong L., Chen Y., Yu Q., Shen M., Xie J. (2023). Purple red rice bran anthocyanins reduce the digestibility of rice starch by forming V-type inclusion complexes. Food Res. Int..

[98] Zhang J., Liu Y., Wang P., Zhao Y., Zhu Y., Xiao X. (2025). The Effect of Protein–Starch Interaction on the Structure and Properties of Starch, and Its Application in Flour Products. Foods.

[99] Chen X., He X., Fu X., Zhang B., Huang Q. (2017). Complexation of rice starch/flour and maize oil through heat moisture treatment: Structural, in vitro digestion and physicochemical properties. Int. J. Biol. Macromol..

[100] Van Ngo T., Kusumawardani S., Kunyanee K., Luangsakul N. (2022). Polyphenol-modified starches and their applications in the food industry: Recent updates and future directions. Foods.

[101] Cagampang G., Cruz L., Espiritu S., Santiago R., Juliano B. (1966). Studies on the Extraction and Composition of Rice Proteins. Cereal Chem..

[102] Zhang Z., Zhang M., Zhao W. (2023). Effect of starch-protein interaction on regulating the digestibility of waxy rice starch under radio frequency treatment with added CaCl2. Int. J. Biol. Macromol..

[103] Khatun A., Waters D.L., Liu L. (2020). The impact of rice protein on in vitro rice starch digestibility. Food Hydrocoll..

[104] Lu X., Ma R., Qiu H., Sun C., Tian Y. (2021). Mechanism of effect of endogenous/exogenous rice protein and its hydrolysates on rice starch digestibility. Int. J. Biol. Macromol..

[105] Ye J., Hu X., Luo S., McClements D.J., Liang L., Liu C. (2018). Effect of endogenous proteins and lipids on starch digestibility in rice flour. Food Res. Int..

[106] Lu X., Chang R., Lu H., Ma R., Qiu L., Tian Y. (2021). Effect of amino acids composing rice protein on rice starch digestibility. LWT.

[107] Chi C., Li X., Zhang Y., Chen L., Li L. (2018). Understanding the mechanism of starch digestion mitigation by rice protein and its enzymatic hydrolysates. Food Hydrocoll..

[108] Kumar A., Panda P.A., Lal M.K., Ngangkham U., Sahu C., Soren K.R., Subudhi H.N., Samantaray S., Sharma S. (2020). Addition of Pulses, Cooking Oils, and Vegetables Enhances Resistant Starch and Lowers the Glycemic Index of Rice (*Oryza sativa* L.). Starch-Starke.

[109] Shen Y., Wu D., Fogliano V., Pellegrini N. (2021). Rice varieties with a high endosperm lipid content have reduced starch digestibility and increased γ-oryzanol bioaccessibility. Food Funct..

[110] Shu X., Jia L., Ye H., Li C., Wu D. (2009). Slow digestion properties of rice different in resistant starch. J. Agric. Food Chem..

[111] Luangsakul N., Ritudomphol O. (2018). Effect of oil addition on in vitro starch digestibility and physicochemical properties of instant rice. Int. J. Agric. Technol..

[112] Fibri D.L.N., Marsono Y. (2024). FiberCreme Addition in Rice Increases the Dietary Fiber, Resistant Starch and Decreases Glycemic Index. Indones. Food Sci. Technol. J..

[113] Pinyo J., Wongsagonsup R., Panthong N., Kantiwong P., Huang Q., Tangsrianugul N., Suphantharika M. (2023). Effects of different edible oils on *in vitro* starch digestibility and physical properties of rice starch and rice flour. Int. J. Food Sci. Technol..

[114] Liu Z., Chen L., Zheng B. (2022). Control of starch–lipid interactions on starch digestibility during hot-extrusion 3D printing for starchy foods. Food Funct..

[115] Mohan V., Anjana R.M., Gayathri R., Bai M.R., Lakshmipriya N., Ruchi V., Balasubramaniyam K.K., Jakir M.M., Shobana S., Unnikrishnan R. (2016). Glycemic Index of a Novel High-Fiber White Rice Variety Developed in India—A Randomized Control Trial Study. Diabetes Technol. Ther..

[116] Veni B.K., Raja D.S., Sridevi P., Tushara M. (2024). Impact of starch profile on glycemic index of coloured and non-pigmented genotypes of rice (*Oryza sativa* L.). Electron. J. Plant Breed..

[117] Chaichoompu E., Ruengphayak S., Wattanavanitchakorn S., Wansuksri R., Yonkoksung U., Suklaew P.O., Chotineeranat S., Raungrusmee S., Vanavichit A., Toojinda T. (2024). Development of Whole-Grain Rice Lines Exhibiting Low and Intermediate Glycemic Index with Decreased Amylose Content. Foods.

[118] Kwaśny D., Borczak B., Kapusta-Duch J., Kron I. (2024). The Influence of Different Polyphenols on the Digestibility of Various Kinds of Starch and the Value of the Estimated Glycemic Index. Appl. Sci..

[119] Al-Atbi D.M., Alhelfi N., Mansour A.A. (2024). Glycemic Index and glycemic load for different types of cooked rice for healthy volunteers. J. Glob. Innov. Agric. Sci..

[120] Yulianto W.A., Suryani C.L., Susiati M., Permana H.I. (2018). Evaluation of Chromium Fortified-Parboiled Rice Coated with Herbal Extracts: Resistant Starch, and Glycemic Index. Int. J. Nutr. Food Eng..

[121] Geng D., Liu L., Lin Z., Zhu L., Deng J., Chen J., Xiang Z., Yao H., Su X., Xia C. (2021). Effects of red lentil protein addition on textural quality and starch digestibility of brown rice noodles. Int. J. Food Sci. Technol..

[122] López-Barón N., Gu Y., Vasanthan T., Hoover R. (2017). Plant proteins mitigate in vitro wheat starch digestibility. Food Hydrocoll..

[123] Ai Y., Jane J.-L. (2024). Understanding starch structure and functionality. Starch in Food.

[124] Gan L., Huang B., Song Z., Zhang Y., Zhang Y., Chen S., Tong L., Wei Z., Yu L., Luo X. (2021). Unique glutelin expression patterns and seed endosperm structure facilitate glutelin accumulation in polyploid rice seed. Rice.

[125] Teo C., Karim A.A., Cheah P., Norziah M., Seow C. (2000). On the roles of protein and starch in the aging of non-waxy rice flour. Food Chem..

[126] López-Barón N., Sagnelli D., Blennow A., Holse M., Gao J., Saaby L., Müllertz A., Jespersen B., Vasanthan T. (2018). Hydrolysed pea proteins mitigate in vitro wheat starch digestibility. Food Hydrocoll..

[127] Wu C., Gong X., Zhang J., Zhang C., Qian J.-Y., Zhu W. (2023). Effect of rice protein on the gelatinization and retrogradation properties of rice starch. Int. J. Biol. Macromol..

[128] Baxter G., Blanchard C., Zhao J. (2014). Effects of glutelin and globulin on the physicochemical properties of rice starch and flour. J. Cereal Sci..

[129] Zhang C., Xu Z., Xu Y., Ma M., Xu S., Gebre B.A., Corke H., Sui Z. (2024). Absolute Quantitative Lipidomics Reveals Different Granule-Associated Surface Lipid Roles in the Digestibility and Pasting of Waxy, Normal, and High-Amylose Rice Starches. J. Agric. Food Chem..

[130] Zhao X., Mei T., Cui B. (2024). Lipids-modified starch: Advances in structural characteristic, physicochemical property, and application. Food Res. Int..

[131] Kaur B., Ranawana V., Teh A., Henry C.J. (2015). The glycemic potential of white and red rice affected by oil type and time of addition. J. Food Sci..

[132] Farooq A.M., Dhital S., Li C., Zhang B., Huang Q. (2018). Effects of palm oil on structural and in vitro digestion properties of cooked rice starches. Int. J. Biol. Macromol..

[133] Zu-Man D., Yu-Long Z., Chun-Yang T., Chuang L., Jia-Qin F., Qiang H., Chun C., Li-Jun Y., Chin-Ping T., Hui N. (2024). Construction of blackberry polysaccharide nano-selenium particles: Structure features and regulation effects of glucose/lipid metabolism in HepG2 cells. Food Res. Int..

[134] Niu H., Chen X., Fu X., Zhang B., Dou Z., Huang Q. (2025). Pectin-stabilized emulsions: Structure-emulsification relationships, covalent and non-covalent modifications, and future trends. Trends Food Sci. Technol..

[135] Liu X., Zhao J., Zhang X., Li Y., Zhao J., Li T., Zhou B., Yang H., Qiao L. (2018). Enrichment of soybean dietary fiber and protein fortified rice grain by dry flour extrusion cooking: The physicochemical, pasting, taste, palatability, cooking and starch digestibility properties. RSC Adv..

[136] Zhang H., Sun S., Ai L. (2022). Physical barrier effects of dietary fibers on lowering starch digestibility. Curr. Opin. Food Sci..

[137] Zhuang J., Zhu J., Cheung P.C., Li C. (2024). The physical and chemical interactions between starch and dietary fiber: Their impact on the physicochemical and nutritional properties of starch. Trends Food Sci. Technol..

[138] Cosme P., Rodríguez A.B., Espino J., Garrido M. (2020). Plant phenolics: Bioavailability as a key determinant of their potential health-promoting applications. Antioxidants.

[139] Liu M., Hu B., Zhang H., Zhang Y., Wang L., Qian H., Qi X. (2017). Inhibition study of red rice polyphenols on pancreatic α-amylase activity by kinetic analysis and molecular docking. J. Cereal Sci..

[140] Aalim H., Luo Z. (2021). Insight into rice (*Oryza sativa* L.) cooking: Phenolic composition, inhibition of α-amylase and α-glucosidase, and starch physicochemical and functional properties. Food Biosci..

[141] Chusak C., Ying J.A.Y., Zhien J.L., Pasukamonset P., Henry C.J., Ngamukote S., Adisakwattana S. (2019). Impact of *Clitoria ternatea* (butterfly pea) flower on in vitro starch digestibility, texture and sensory attributes of cooked rice using domestic cooking methods. Food Chem..

[142] El Oirdi M. (2024). Harnessing the Power of Polyphenols: A New Frontier in Disease Prevention and Therapy. Pharmaceuticals.

[143] Tai N., Kunyanee K., Luangsakul N. (2023). Multivariable analysis of physicochemical and functional charac-terization of four Thai pigmented rice varieties. Int. J. Agric. Technol..

[144] Yamuangmorn S., Prom-u-Thai C. (2021). The potential of high-anthocyanin purple rice as a functional ingredient in human health. Antioxidants.

[145] Yang J., Dong M., Fang F., Li Y., Li C. (2024). Effects of varied preparation processes on polyphenol-rice starch complexes, in vitro starch digestion, and polyphenols release. Food Chem..

[146] Han X., Zhang M., Zhang R., Huang L., Jia X., Huang F., Liu L. (2020). Physicochemical interactions between rice starch and different polyphenols and structural characterization of their complexes. LWT.

[147] Jakobek L. (2015). Interactions of polyphenols with carbohydrates, lipids and proteins. Food Chem..

[148] Jeon J.-S., Ryoo N., Hahn T.-R., Walia H., Nakamura Y. (2010). Starch biosynthesis in cereal endosperm. Plant Physiol. Biochem..

[149] Cakir B., Tian L., Crofts N., Chou H., Koper K., Ng C., Tuncel A., Gargouri M., Hwang S., Fujita N. (2019). Re-programming of gene expression in the CS8 rice line over-expressing ADP glucose pyrophosphorylase induces a suppressor of starch biosynthesis. Plant J..

[150] Yang R., Bai J., Fang J., Wang Y., Lee G., Piao Z. (2016). A single amino acid mutation of OsSBEIIb contributes to resistant starch accumulation in rice. Breed. Sci..

[151] Bao J., Zhou X., Xu F., He Q., Park Y. (2017). Genome-wide association study of the resistant starch content in rice grains. Starch-Starke.

[152] Kong X., Chen Y., Zhu P., Sui Z., Corke H., Bao J. (2015). Relationships among genetic, structural, and functional properties of rice starch. J. Agric. Food Chem..

[153] Zhou H., Wang L., Liu G., Meng X., Jing Y., Shu X., Kong X., Sun J., Yu H., Smith S.M. (2016). Critical roles of soluble starch synthase SSIIIa and granule-bound starch synthase Waxy in synthesizing resistant starch in rice. Proc. Natl. Acad. Sci. USA.

[154] Tsuiki K., Fujisawa H., Itoh A., Sato M., Fujita N. (2016). Alterations of starch structure lead to increased resistant starch of steamed rice: Identification of high resistant starch rice lines. J. Cereal Sci..

[155] Badoni S., Pasion-Uy E.A., Kor S., Kim S.-R., Tiozon R.N., Misra G., Buenafe R.J.Q., Labarga L.M., Ramos-Castrosanto A.R., Pratap V. (2024). Multiomics of a rice population identifies genes and genomic regions that bestow low glycemic index and high protein content. Proc. Natl. Acad. Sci. USA.

[156] Kongkachuichai R., Charoensiri R., Meekhruerod A., Kettawan A. (2020). Effect of processing conditions on bioactive compounds and glycemic index of the selected landrace rice variety in pre-diabetes. J. Cereal Sci..

[157] Wang Z., Zhang M., Liu G., Deng Y., Zhang Y., Tang X., Li P., Wei Z. (2021). Effect of the degree of milling on the physicochemical properties, pasting properties and in vitro digestibility of *Simiao* rice. Grain Oil Sci. Technol..

[158] Tian Y., Ding L., Liu Y., Shi L., Wang T., Wang X., Dang B., Li L., Gou G., Wu G. (2023). The Effect of Different Milling Methods on the Physicochemical and In Vitro Digestibility of Rice Flour. Foods.

[159] Shobana S., Lakshmipriya N., Bai M.R., Gayathri R., Ruchi V., Sudha V., Malleshi N.G., Krishnaswamy K., Henry C.-J.K., Anjana R.M. (2017). Even minimal polishing of an Indian parboiled brown rice variety leads to increased glycemic responses. Asia Pac. J. Clin. Nutr..

[160] Yang W., Zheng Y., Sun W., Chen S., Liu D., Zhang H., Fang H., Tian J., Ye X. (2020). Effect of extrusion processing on the microstructure and in vitro digestibility of broken rice. LWT.

[161] Cai C., Tian Y., Yu Z., Sun C., Jin Z. (2020). In Vitro Digestibility and Predicted Glycemic Index of Chemically Modified Rice Starch by One-Step Reactive Extrusion. Starch-Starke.

[162] Naseer B., Naik H.R., Hussain S.Z., Zargar I., Beenish, Bhat T.A., Nazir N. (2021). Effect of carboxymethyl cellulose and baking conditions on in-vitro starch digestibility and physico-textural characteristics of low glycemic index gluten-free rice cookies. LWT.

[163] Bagchi T.B., Das B., Kumar A., Kumar G., Banerjee J., Gain H., Adhikari A.A., Chattopadhyay K. (2023). Impact of cooking, parboiling and fermentation on nutritional components, predicted glycemic index and pasting properties of rice. J. Cereal Sci..

[164] Garg S., Sharma N., Kumari A., Bala M., Kaur R. (2024). Impact of parboiling on nutritionally important starch fractions, pasting properties, and in vitro starch digestibility of rice genotypes. Cereal Res. Commun..

[165] Soltani A., Golmakani M.-T., Fazaeli M., Niakousari M., Hosseini S.M.H. (2023). Evaluating the effect of different physical pretreatments and cooking methods on nutritional (starch digestibility) and physicochemical properties of white rice grains (Fajr cultivar). LWT.

[166] Sanusi M., Hussein J. (2023). Impacts of soaking time and steaming time on proximate, vitro-starch digestibility and amylose content of short, medium and long rice grain type. Carpathian J. Food Sci. Technol..

[167] Yang R., Tang J., Zhao Q., Piao Z., Lee G., Wan C., Bai J. (2023). Starch Properties of Roasting Rice from Naturally High-Resistant Starch Rice Varieties. Molecules.

[168] Karupaiah T., Aik C.K., Heen T.C., Subramaniam S., Bhuiyan A.R., Fasahat P., Zain A.M., Ratnam W. (2011). A transgressive brown rice mediates favourable glycaemic and insulin responses. J. Sci. Food Agric..

[169] Casiraghi M., Brighenti F., Pellegrini N., Leopardi E., Testolin G. (1993). Effect of Processing on Rice Starch Digestibility Evaluated by in Vivo and in Vitro Methods. J. Cereal Sci..

[170] Mohan V., Spiegelman D., Sudha V., Gayathri R., Hong B., Praseena K., Anjana R.M., Wedick N.M., Arumu-gam K., Malik V. (2014). Effect of brown rice, white rice, and brown rice with legumes on blood glucose and in-sulin responses in overweight Asian Indians: A randomized controlled trial. Diabetes Technol. Ther..

[171] Sasaki T., Okunishi T., Sotome I., Okadome H. (2016). Effects of milling and cooking conditions of rice on in vitro starch digestibility and blood glucose response. Cereal Chem..

[172] Li F., Guan X., Li C. (2021). Effects of degree of milling on the starch digestibility of cooked rice during (in vitro) small intestine digestion. Int. J. Biol. Macromol..

[173] Chanvrier H., Pillin C.N., Vandeputte G., Haiduc A., Leloup V., Gumy J.-C. (2015). Impact of extrusion parameters on the properties of rice products: A physicochemical and X-ray tomography study. Food Struct..

[174] Das A.B., Bhattacharya S. (2019). Characterization of the batter and gluten-free cake from extruded red rice flour. LWT.

[175] Goger A., Thompson M., Pawlak J., Arnould M., Klymachyov A., Sheppard R., Lawton D. (2018). Inline rheological behavior of dispersed water in a polyester matrix with a twin screw extruder. Polym. Eng. Sci..

[176] Sivakamasundari S.K., Priyanga S., Moses J.A., Anandharamakrishnan C. (2021). Impact of processing techniques on the glycemic index of rice. Crit. Rev. Food Sci. Nutr..

[177] Feng Y., Lee Y. (2014). Effect of specific mechanical energy on in-vitro digestion and physical properties of extruded rice-based snacks. Food Nutr. Sci..

[178] Zeng Z., Huang K., McClements D.J., Hu X., Luo S., Liu C. (2019). Phenolics, antioxidant activity, and in vitro starch digestibility of extruded brown rice influenced by Choerospondias axillaris fruit peels addition. Starch-Stärke.

[179] Ye J., Liu C., Luo S., Wu J., Hu X., McClements D.J. (2019). A simulated gastrointestinal tract study of texturized rice grains: Impact of texturization on starch digestibility. J. Cereal Sci..

[180] Giuberti G., Marti A., Fortunati P., Gallo A. (2017). Gluten free rice cookies with resistant starch ingredients from modified waxy rice starches: Nutritional aspects and textural characteristics. J. Cereal Sci..

[181] Rakmai J., Haruthaithanasan V., Chompreeda P., Chatakanonda P., Yonkoksung U. (2021). Development of gluten-free and low glycemic index rice pancake: Impact of dietary fiber and low-calorie sweeteners on texture pro-file, sensory properties, and glycemic index. Food Hydrocoll. Health.

[182] Thakur N., Raigond P., Singh Y., Mishra T., Singh B., Lal M.K., Dutt S. (2020). Recent updates on bioaccessi-bility of phytonutrients. Trends Food Sci. Technol..

[183] Singh A., Raigond P., Lal M.K., Singh B., Thakur N., Changan S.S., Kumar D., Dutt S. (2020). Effect of cooking methods on glycemic index and in vitro bioaccessibility of potato (*Solanum tuberosum* L.) carbohydrates. LWT.

[184] Jung E.Y., Suh H.J., Hong W.S., Kim D.G., Hong Y.H., Hong I.S., Chang U.J. (2009). Uncooked rice of relatively low gelatinization degree resulted in lower metabolic glucose and insulin responses compared with cooked rice in female college students. Nutr. Res..

[185] Wolever T.M.S., Vorster H.H., Björck I., Brand-Miller J., Brighenti F., Mann J.I., Ramdath D.D., Granfeldt Y., Holt S., Perry T.L. (2003). Determination of the glycaemic index of foods: Interlaboratory study. Eur. J. Clin. Nutr..

[186] Liu X., Huang S., Chao C., Yu J., Copeland L., Wang S. (2022). Changes of starch during thermal processing of foods: Current status and future directions. Trends Food Sci. Technol..

[187] Mohamed I.O. (2021). Effects of processing and additives on starch physicochemical and digestibility properties. Carbohydr. Polym. Technol. Appl..

[188] Fan C., Cheng L., Hong Y., Li Z., Li C., Ban X., Gu Z. (2024). Study on the gelatinization and digestive characteristics of wheat starch and potato starch under low moisture conditions. Int. J. Biol. Macromol..

[189] Kumari A., Roy A. (2024). Impact of the degree of starch gelatinization on the texture, soaking, and cooking characteristics of high amylose rice: An experimental and numerical study. J. Food Meas. Charact..

[190] Shukla A.P., Iliescu R.G., Thomas C.E., Aronne L.J. (2015). Food order has a significant impact on postpran-dial glucose and insulin levels. Diabetes Care.

[191] Sagum R., Arcot J. (2000). Effect of domestic processing methods on the starch, non-starch polysaccharides and in vitro starch and protein digestibility of three varieties of rice with varying levels of amylose. Food Chem..

[192] Ai Y., Hasjim J., Jane J.-L. (2013). Effects of lipids on enzymatic hydrolysis and physical properties of starch. Carbohydr. Polym..

[193] Gunathilaka M., Ekanayake S. (2015). Effect of different cooking methods on glycaemic index of Indian and Pakistani basmati rice varieties. Ceylon Med J..

[194] Ritudomphol O., Luangsakul N. (2019). Optimization of processing condition of instant rice to lower the glycemic index. J. Food Sci..

[195] Kim H.R., Hong J.S., Ryu A., Choi H. (2020). Combination of rice varieties and cooking methods resulting in a high content of resistant starch. Cereal Chem..

[196] Wang L., Zhao S., Kong J., Li N., Qiao D., Zhang B., Xu Y., Jia C. (2020). Changing cooking mode can slow the starch digestion of colored brown rice: A view of starch structural changes during cooking. Int. J. Biol. Macromol..

[197] Kwofie E., Ngadi M. (2017). A review of rice parboiling systems, energy supply, and consumption. Renew. Sustain. Energy Rev..

[198] Buggenhout J., Brijs K., Celus I., Delcour J. (2013). The breakage susceptibility of raw and parboiled rice: A re-view. J. Food Eng..

[199] Muchlisyiyah J., Shamsudin R., Kadir Basha R., Shukri R., How S., Niranjan K., Onwude D. (2023). Parboiled Rice Processing Method, Rice Quality, Health Benefits, Environment, and Future Perspectives: A Review. Agriculture.

[200] Scazzina F., Dall’asta M., Casiraghi M., Sieri S., Del Rio D., Pellegrini N., Brighenti F. (2016). Glycemic index and glycemic load of commercial Italian foods. Nutr. Metab. Cardiovasc. Dis..

[201] Rondanelli M., Haxhari F., Gasparri C., Barrile G.C., Cavioni A., Guido D., Mansueto F., Zese M., Mazzola G., Moroni A. (2023). Glycemic Index and Amylose Content of 25 Japonica Rice Italian Cultivar. Starch-Starke.

[202] Pathiraje P., Madhujith W., Chandrasekara A., Nissanka S. (2011). The effect of rice variety and parboiling on in vivo glycemic response. Trop. Agric. Res..

[203] Bhar S., Bose T., Dutta A., Mande S.S. (2022). A perspective on the benefits of consumption of parboiled rice over brown rice for glycaemic control. Eur. J. Nutr..

[204] Chakraborty I., N. P., Mal S.S., Paul U.C., Rahman H., Mazumder N. (2022). An insight into the gelatinization properties influencing the modified starches used in food industry: A review. Food Bioprocess Technol..

[205] Chang Q., Zheng B., Zhang Y., Zeng H. (2021). A comprehensive review of the factors influencing the formation of retrograded starch. Int. J. Biol. Macromol..

[206] Chung H.-J., Lim H.S., Lim S.-T. (2006). Effect of partial gelatinization and retrogradation on the enzymatic di-gestion of waxy rice starch. J. Cereal Sci..

[207] Kim J., Kim W., Shin M. (1997). A Comparative study on retrogradation of rice starch gels by dsc, x-ray and α-amylase methods. Starch-Starke.

[208] Chakraborty I., Govindaraju I., Kunnel S., Managuli V., Mazumder N. (2023). Effect of Storage Time and Tem-perature on Digestibility, Thermal, and Rheological Properties of Retrograded Rice. Gels.

